# Differential global distribution of marine picocyanobacteria gene clusters reveals distinct niche-related adaptive strategies

**DOI:** 10.1038/s41396-023-01386-0

**Published:** 2023-02-25

**Authors:** Hugo Doré, Ulysse Guyet, Jade Leconte, Gregory K. Farrant, Benjamin Alric, Morgane Ratin, Martin Ostrowski, Mathilde Ferrieux, Loraine Brillet-Guéguen, Mark Hoebeke, Jukka Siltanen, Gildas Le Corguillé, Erwan Corre, Patrick Wincker, David J. Scanlan, Damien Eveillard, Frédéric Partensky, Laurence Garczarek

**Affiliations:** 1 Sorbonne Université, CNRS, UMR 7144 Adaptation and Diversity in the Marine Environment (AD2M), Station Biologique de Roscoff (SBR), Roscoff, France; 2 School of Life Sciences, University of Warwick, Coventry, CV4 7AL UK; 3 CNRS, FR 2424, ABiMS Platform, Station Biologique de Roscoff (SBR), Roscoff, France; 4 Sorbonne Université, CNRS, UMR 8227, Integrative Biology of Marine Models (LBI2M), Station Biologique de Roscoff (SBR), Roscoff, France; 5 Genoscope, Institut de biologie François-Jacob, Commissariat à l’Energie Atomique (CEA), Université Paris-Saclay, Evry, France; 6 Génomique Métabolique, Genoscope, Institut de biologie François Jacob, CEA, CNRS, Université d’Evry, Université Paris-Saclay, Evry, France; 7 Research Federation (FR2022) Tara Océans GO-SEE, Paris, France; 8 Nantes Université, Centrale Nantes, CNRS, LS2N, UMR 6004, Nantes, France; 9 Present Address: Climate Change Cluster, University of Technology, Broadway, NSW 2007 Australia

**Keywords:** Water microbiology, Microbial ecology

## Abstract

The ever-increasing number of available microbial genomes and metagenomes provides new opportunities to investigate the links between niche partitioning and genome evolution in the ocean, especially for the abundant and ubiquitous marine picocyanobacteria *Prochlorococcus* and *Synechococcus*. Here, by combining metagenome analyses of the *Tara* Oceans dataset with comparative genomics, including phyletic patterns and genomic context of individual genes from 256 reference genomes, we show that picocyanobacterial communities thriving in different niches possess distinct gene repertoires. We also identify clusters of adjacent genes that display specific distribution patterns in the field (eCAGs) and are thus potentially involved in the same metabolic pathway and may have a key role in niche adaptation. Several eCAGs are likely involved in the uptake or incorporation of complex organic forms of nutrients, such as guanidine, cyanate, cyanide, pyrimidine, or phosphonates, which might be either directly used by cells, for example for the biosynthesis of proteins or DNA, or degraded to inorganic nitrogen and/or phosphorus forms. We also highlight the enrichment of eCAGs involved in polysaccharide capsule biosynthesis in *Synechococcus* populations thriving in both nitrogen- and phosphorus-depleted areas vs. low-iron (Fe) regions, suggesting that the complexes they encode may be too energy-consuming for picocyanobacteria thriving in the latter areas. In contrast, *Prochlorococcus* populations thriving in Fe-depleted areas specifically possess an alternative respiratory terminal oxidase, potentially involved in the reduction of Fe(III) to Fe(II). Altogether, this study provides insights into how phytoplankton communities populate oceanic ecosystems, which is relevant to understanding their capacity to respond to ongoing climate change.

## Introduction

Although phytoplankton communities play a crucial role in marine biogeochemical cycles [1, 2], the relative contribution of different species or ecotypes to these cycles remains difficult to assess due to a lack of knowledge of specific metabolic traits. Indeed, trait-based functional diversity is thought to be a better predictor of ecosystem functioning than species diversity [3, 4], and understanding which metabolic traits have facilitated the adaptation of an ecotype to a particular environment is key to understanding each species’ ecological role. Comparative genomics has sometimes been used to try to identify the genetic basis of niche adaptation. However, this approach has revealed only a few genes specific to particular ecotypes and thus potentially involved in niche adaptation, perhaps due to the fairly low number of genomes available (even for major phytoplankton groups) and the poor ecological representation and physiological characterization of available isolates [5–9]. An alternative to better deciphering the links between functional diversity and niche partitioning involves exploiting the rapidly growing number of metagenomes. These can be used to generate metagenome-assembled genomes (MAGs) that can fill the gaps for yet uncultured microbial taxa as well as to identify niche-specific genes, i.e. genes enriched in specific spatial and/or temporal environmental conditions, by recruiting metagenomic reads onto reference genomes [10–16].

Due to their abundance and ubiquity in the field and the large number of available genomes, including single amplified genomes (SAGs) and MAGs [6, 17–20], the marine picocyanobacteria *Prochlorococcus* and *Synechococcus* constitute highly pertinent models to study how phytoplankton cells adapt to their variable physico-chemical environment. These genera are indeed the two most abundant members of the phytoplankton community, *Prochlorococcus* being restricted to the 40°S-50°N latitudinal band, whilst the distribution of *Synechococcus* extends from the equator to subpolar waters [21–23]. By combining laboratory and environmental studies, scientists have managed to decipher their genetic diversity and delineate specific ecotypes or “ecologically significant taxonomic units” (ESTUs), i.e. genetic groups within clades occupying a specific ecological niche [24–28]. While three major ESTU assemblages were identified for *Prochlorococcus* in surface waters, whose distribution was found to be mainly driven by temperature and iron (Fe) availability, eight distinct assemblages were identified for *Synechococcus* depending on three main environmental parameters: temperature, Fe, and phosphorus (P) availability. Nevertheless, few studies have so far linked knowledge of the distribution of the different ecotypes to their functional diversity in order to identify potential niche-specific genes, based on gene relative abundance in different ecosystems [10, 14, 15, 29, 30]. Furthermore, most of these previous studies have focused on the abundance of individual genes, or more rarely, on just a few genomic regions with known function, for example those involved in nitrogen (N) or P uptake and assimilation [31–33].

Here, in order to better understand the relationship between biogeochemistry and metabolic traits of marine picocyanobacteria, we searched for global patterns of picocyanobacterial gene distributions. To do so, we used a network approach to integrate metagenome analyses of the oceanwide *Tara* Oceans dataset and synteny of individual accessory genes in 256 reference genomes, SAGs, or MAGs covering the wide diversity existing within *Prochlorococcus* and *Synechococcus*. Using this approach, we identified many clusters of adjacent genes that display distinctive global distribution patterns in situ and thus likely play important roles in the adaptation of these bacteria to the main ecological niches they occupy in the ocean. Given that gene synteny is commonly used as an indicator of shared function [34, 35], delineation of these gene clusters should also help to identify the putative function of numerous unknown genes, based on their occurrence alongside functionally annotated genes in the same cluster. Overall, this study provides novel insights into the genetic basis of niche partitioning in key members of the phytoplankton community.

## Results and discussion

### Different picocyanobacterial communities exhibit distinct gene repertoires

**Fig. 1:**
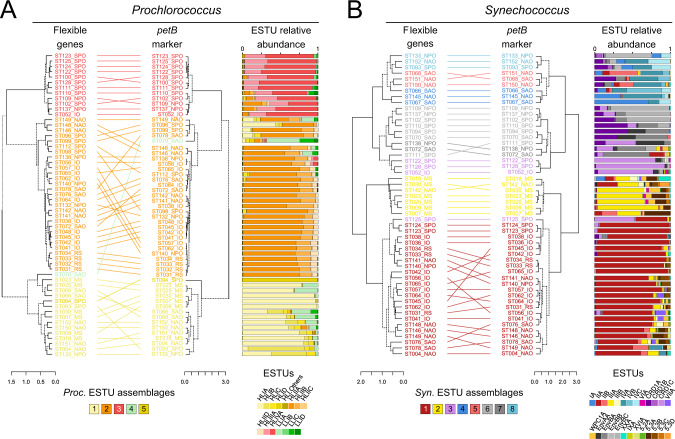
Comparison of clustering based on relative abundance profiles of ecologically significant taxonomic units (ESTUs) and of flexible genes for both picocyanobacteria. **A** *Prochlorococcus*. **B** *Synechococcus*. Leaves of the trees correspond to stations along the *Tara* Oceans transect that are colored according to the code shown at the bottom of the trees, which corresponds to ESTU assemblages as determined previously [24] by clustering stations exhibiting similar ESTU relative abundance profiles shown here on the right of each tree (for global distribution maps of ESTU assemblages, see Figs. 3B and 4B in [24]). ESTUs were colored according to the palette below each panel. Dotted lines in dendrograms indicate discrepancies between tree topologies. Accessory genes correspond to all genes except those defined as large-core genes in a previous study [6]. Of note, due to a slightly different clustering method (cf. materials and methods), assemblage 7 (dark grey stations in 1B), which was discriminated from assemblage 6 in the Farrant et al. (2016) now clusters with this assemblage. Abbreviations: IO Indian Ocean, MS Mediterranean Sea, NAO North Atlantic Ocean, NPO North Pacific Ocean, RS Red Sea, SAO South Atlantic Ocean, SO Southern Ocean.

To analyze the distribution of *Prochlorococcus* and *Synechococcus* reads along the *Tara* Oceans transect, metagenomic reads corresponding to the bacterial size fraction were recruited against 256 picocyanobacterial reference genomes, including SAGs and MAGs representative of uncultured lineages (e.g., *Prochlorococcus* HLIII-IV, *Synechococcus* EnvA or EnvB). This yielded a total of 1.07 billion recruited reads, of which 87.7% mapped onto *Prochlorococcus* genomes and 12.3% onto *Synechococcus* genomes, which were then functionally assigned by mapping them onto the manually curated Cyanorak *v2.1* CLOG database [19]. In order to identify picocyanobacterial genes potentially involved in niche adaptation, we analyzed the distribution across the oceans of flexible (i.e. non-core) genes. Clustering of *Tara* Oceans stations according to the relative abundance of flexible genes resulted in three well-defined clusters for *Prochlorococcus* (Fig. 1A), which matched those obtained when stations were clustered according to the relative abundance of *Prochlorococcus* ESTUs, as assessed using the high-resolution marker gene *petB*, encoding cytochrome *b*_6_ (Fig. 1A; [24]). Only a few discrepancies can be observed between the two trees, including stations TARA-070 that displayed one of the most disparate ESTU compositions and TARA-094, dominated by the rare HLID ESTU (Fig. 1A). Similarly, for *Synechococcus*, most of the eight assemblages of stations discriminated based on the relative abundance of ESTUs (Fig. 1B) were also retrieved in the clustering based on flexible gene abundance, except for a few intra-assemblage switches between stations, notably those dominated by ESTU IIA (Fig. 1B). Despite these few variations, four major clusters can be clearly delineated in both *Synechococcus* trees, corresponding to four broadly defined ecological niches, namely (i) cold, nutrient-rich, pelagic or coastal environments (blue and light red in Fig. 1B), (ii) Fe-limited environments (purple and grey), (iii) temperate, P-depleted, Fe-replete areas (yellow) and (iv) warm, N-depleted, Fe-replete regions (dark red). This correspondence between taxonomic and functional information was also confirmed by the high congruence between distance matrices based on ESTU relative abundance and on CLOG relative abundance (*p*-value < 10^−4^, mantel test *r* = 0.84 and *r* = 0.75 for *Synechococcus* and *Prochlorococcus*, respectively; dataset 1–4). Altogether, this indicates that distinct picocyanobacterial communities, as assessed based on a single taxonomic marker, also display different gene repertoires. As previously suggested for *Prochlorococcus* [36], this strong correlation between taxonomy and gene content strengthens the idea that, in both genera, the evolution of the accessory genome mainly occurs by vertical transmission, with a relatively low extent of lateral gene transfers, although we cannot exclude that the latter events may occur more often within members of a given ecotype.

### Distribution of flexible genes is tightly linked to environmental parameters and ESTUs

**Fig. 2:**
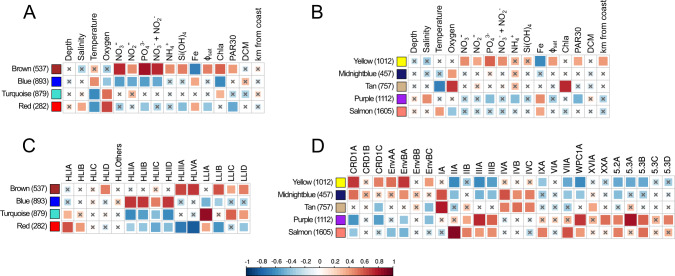
Correlation of picocyanobacterial module eigengenes to physico-chemical parameters and ESTU abundance. **A**, **B** Correlation of module eigengenes to physico-chemical parameters for *Prochlorococcus* (**A**) and *Synechococcus* (**B**). **C**, **D** Correlation of module eigengenes to relative abundance profiles of ESTUs *sensu* [4]. Pearson (**A**, **B**) and Spearman (**C**, **D**) correlation coefficients (*r* and rho, respectively) are indicated by the color scale. Each module is identified by a specific color and the number between brackets specifies the number of genes in each module. The *eigengene* is representative of the relative abundance of genes of a given module at each *Tara* Oceans station. Non-significant correlations (Student asymptotic *p*-value > 0.01) are marked by a cross. Φsat: index of iron limitation derived from satellite data. PAR30: satellite-derived photosynthetically available radiation at the surface, averaged on 30 days. DCM: depth of the deep chlorophyll maximum.

In order to reduce the amount of data and better interpret the global distribution of picocyanobacterial gene content, a correlation network of genes was built for each genus based on relative abundance profiles of genes across *Tara* Oceans samples using Weighted Correlation Network Analysis (WGCNA). This analysis emphasized four main modules of genes for *Prochlorococcus* (Fig. S1A) and five for *Synechococcus* (Fig. S1B), each module being abundant in a different set of stations. These modules were then associated with the available environmental parameters (Fig. 2A, B) and to the relative abundance of *Prochlorococcus* or *Synechococcus* ESTUs at each station (Fig. 2C, D). For instance, the *Prochlorococcus brown* module was strongly correlated with nutrient concentrations, particularly nitrate and phosphate, and strongly anti-correlated with Fe availability (Fig. 2A). This module thus corresponds to genes preferentially found in Fe-limited high-nutrient low-chlorophyll (HNLC) areas. Indeed, the *brown* module *eigengene* (Fig. S1A), i.e. the first principal component of gene abundances at the different stations for this module, representative of the abundance profiles of genes for this module at the different stations, showed the highest abundances at stations TARA-100 to 125, localized in the South and North Pacific Ocean, as well as at TARA-052, a station located close to the northern coast of Madagascar, likely influenced by the Indonesian throughflow originating from the tropical Pacific Ocean [24, 37]. Furthermore, the correlation of the *Prochlorococcus brown* module with the relative abundance of ESTUs at each station showed that it is also strongly associated with the presence of HLIIIA and HLIVA (Fig. 2C), previously shown to constitute the dominant *Prochlorococcus* ESTUs in low-Fe environments [24, 38, 39] but also the LLIB ESTU, found to dominate the LLI population in these low-Fe areas [24]. Altogether, this example and analyses of all other *Prochlorococcus* and *Synechococcus* modules (supplementary text) show that the communities colonizing cold, Fe-, N-, and/or P-depleted niches possess specific gene repertoires potentially involved in their adaptation to these particular environmental conditions.

### Identification of individual genes potentially involved in niche partitioning

**Fig. 3:**
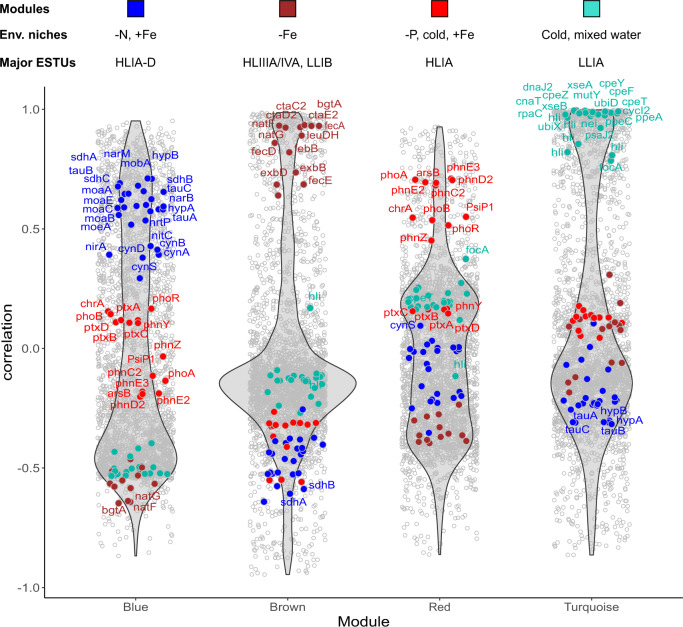
Violin plots highlighting the most representative genes of each *Prochlorococcus* module. For each module, each gene is represented as a dot positioned according to its correlation with the eigengene for each module, the most representative genes being localized on top of each violin plot. Genes mentioned in the text and/or in Dataset 6 have been colored according to the color of the corresponding module, indicated by a colored bar above each module. The text above violin plots indicates the most significant environmental parameter(s) and/or ESTU(s) for each module, as derived from Fig. 2.

To identify genes relevant for adaptation to a specific set of environmental conditions and enriched in specific ESTU assemblages, we selected the most representative genes from each module (Dataset 5; Figs. 3, S2). Most genes retrieved this way encode proteins of unknown or hypothetical function (85.7% of 7,485 genes). However, among the genes with a functional annotation (Dataset 6), a large fraction seems to have a function related to their realized environmental niche (Figs. 3, S2). For instance, many genes involved in the transport and assimilation of nitrite and nitrate (*nirA*, *nirX*, *moaA-C, moaE, mobA, moeA, narB, M, nrtP;* [6]) as well as cyanate, an organic form of nitrogen (*cynA, B, D, S*), are enriched in the *Prochlorococcus blue* module, which is correlated with the HLIIA-D ESTU and to low inorganic N, P, and silica levels and anti-correlated with Fe availability (Fig. 2A–C). This is consistent with previous studies showing that while only a few *Prochlorococcus* strains in culture possess the *nirA* gene and even less the *narB* gene, natural *Prochlorococcus* populations inhabiting N-poor areas do possess one or both of these genes [40–42]. Similarly, numerous genes amongst the most representative of *Prochlorococcus brown, red* and *turquoise* modules are related to adaptation of HLIIIA/IVA, HLIA and LLIA ESTUs to Fe-limited, cold P-limited, and cold, mixed waters, respectively (Fig. 3). Comparable results were obtained for *Synechococcus*, although the niche delineation was less clear than for *Prochlorococcus* since genes within each module exhibited lower correlations with the module *eigenvalue* (Fig. S2). These results therefore constitute a proof of concept that this network analysis was able to retrieve niche-related genes from metagenomics data.

### Identification of eCAGs potentially involved in niche partitioning

**Fig. 4:**
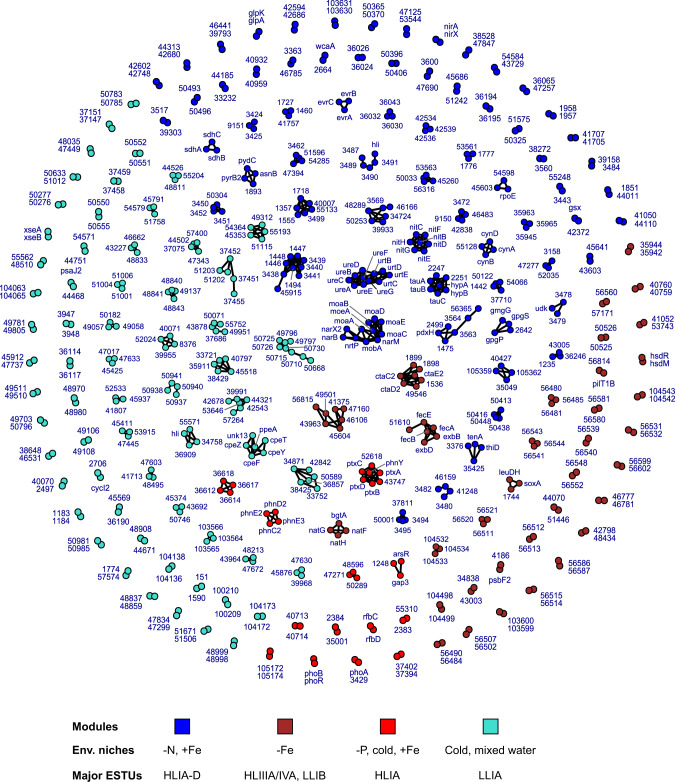
Delineation of *Prochlorococcus* eCAGs, defined as a set of genes that are both adjacent in reference genomes and share a similar in situ distribution. Nodes correspond to individual genes with their gene name (or significant numbers of the CK number, e.g. 1234 for CK_00001234) and are colored according to their WGCNA module. A link between two nodes indicates that these two genes are less than five genes apart in at least one genome. The bottom insert shows the most significant environmental parameter(s) and/or ESTU(s) for each module, as derived from Fig. 2.

In order to better understand the function of niche-related genes, notably of the numerous unknowns, we then integrated global distribution data with gene synteny in reference genomes using a network approach (Datasets 7, 8). This led us to identify clusters of adjacent genes in reference genomes, and thus potentially involved in the same metabolic pathway (Figs. 4, S3, S4; Dataset 6). These clusters were defined within each module and thus encompass genes with similar distribution and abundance in situ. Hereafter, these environmental clusters of adjacent genes will be called “eCAGs”.

#### eCAGs related to nitrogen metabolism

The well-known nitrate/nitrite gene cluster involved in uptake and assimilation of inorganic forms of N (see above), which is present in most *Synechococcus* genomes (Dataset 6), was expectedly not restricted to a particular niche in natural *Synechococcus* populations, as shown by its quasi-absence from WGCNA modules. In *Prochlorococcus*, this cluster is separated into two eCAGs enriched in low-N areas (Fig. S5A, B), most genes being included in Pro-eCAG_002, present in only 13 out of 118 *Prochlorococcus* genomes, while *nirA* and *nirX* form an independent eCAG (Pro-eCAG_001) due to their presence in many more genomes. The quasi-core *ureA-G*/*urtB-E* genomic region was also found to form a *Prochlorococcus* eCAG (Pro-eCAG_003) that was impoverished in low-Fe compared to other regions (Fig. S5C, D), in agreement with its presence in only two out of six HLIII/IV genomes. We also uncovered several other *Prochlorococcus* and *Synechococcus* eCAGs that seem to be involved in the transport and/or assimilation of more unusual and/or complex forms of nitrogen, which might either be degraded into elementary N molecules or possibly directly used by cells for e.g. the biosynthesis of proteins or DNA. Indeed, we detected in both genera an eCAG (Pro-eCAG_004 and Syn-eCAG_001; Fig. S6A, B; Dataset 6) that encompasses *speB2*, an ortholog of *Synechocystis* PCC 6803 *sll1077*, previously annotated as encoding an agmatinase [29, 43] and which was recently characterized as a guanidinase that degrades guanidine rather than agmatine to urea and ammonium [44]. *E. coli* produces guanidine under nutrient-poor conditions, suggesting that guanidine metabolism is biologically significant and potentially prevalent in natural environments [44, 45]. Furthermore, the *ykkC* riboswitch candidate, which was shown to specifically sense guanidine and to control the expression of a variety of genes involved in either guanidine metabolism or nitrate, sulfate, or bicarbonate transport, is located immediately upstream of this eCAG in *Synechococcus* reference genomes, all genes of this cluster being predicted by RegPrecise 3.0 to be regulated by this riboswitch (Fig. S6C; [45, 46]). The presence of *hypA* and *B* homologs within this eCAG furthermore suggests that, in the presence of guanidine, these homologs could be involved in the insertion of Ni_2_^+^, or another metal cofactor, in the active site of guanidinase. The next three genes of this eCAG, which encode an ABC transporter similar to the TauABC taurine transporter in *E. coli* (Fig. S6C), could be involved in guanidine transport in low-N areas. Of note, the presence in most *Synechococcus*/*Cyanobium* genomes possessing this eCAG of a gene encoding a putative Rieske Fe-sulfur protein (CK_00002251) downstream of this gene cluster, seems to constitute a specificity compared to the homologous gene cluster in *Synechocystis* sp. PCC 6803. The presence of this Fe-S protein suggests that Fe is used as a cofactor in this system and might explain why this gene cluster is absent from picocyanobacteria thriving in low-Fe areas, while it is present in a large proportion of the population in most other oceanic areas (Fig. S6A, B).

**Fig. 5:**
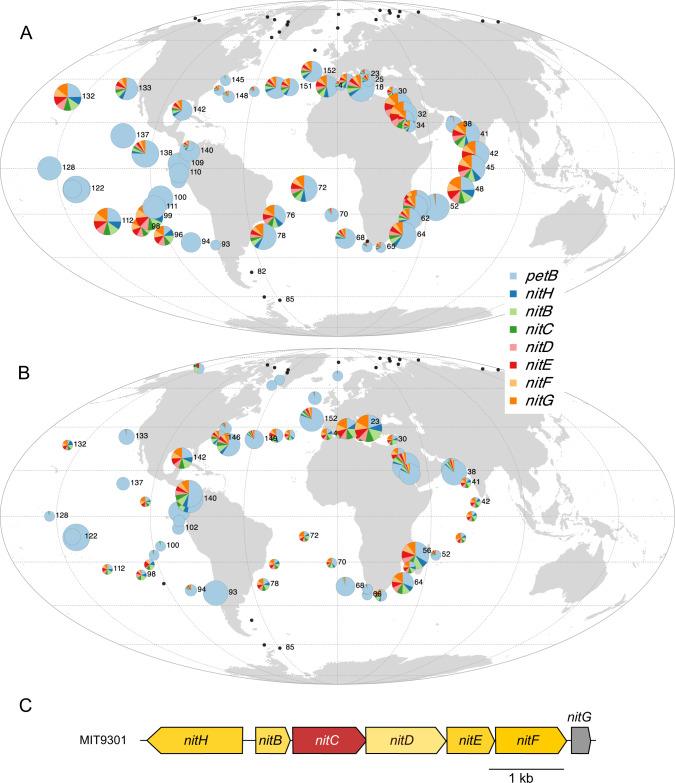
Global distribution map of the eCAG involved in nitrile or cyanide transport and assimilation. **A** *Prochlorococcus* Pro-eCAG_006. **B** *Synechococcus* Syn-eCAG_002. **C** The genomic region in *Prochlorococcus marinus* MIT9301. The size of the circle is proportional to relative abundance of each genus as estimated based on the single-copy core gene *petB* and this gene was also used to estimate the relative abundance of other genes in the population. Black dots represent *Tara* Oceans stations for which *Prochlorococcus* or *Synechococcus* read abundance was too low to reach the threshold limit.

Another example of the use of organic N forms concerns compounds containing a cyano radical (C ≡ N). The cyanate transporter genes (*cynABD*) were indeed found in a *Prochlorococcus* eCAG (Pro-eCAG_005, also including the conserved hypothetical gene CK_00055128; Fig. S7A, B). While only a small proportion of the *Prochlorococcus* community possesses this eCAG in warm, Fe-replete waters, it is absent from other oceanic areas in accordance with its low frequency in *Prochlorococcus* genomes (present in only two HLI and five HLII genomes). In *Synechococcus* these genes were not included in a module, and thus are not in an eCAG (Dataset 6; Fig. S7C), but seem widely distributed despite their presence in only a few *Synechococcus* genomes (mostly in clade III strains; [6, 47, 48]). Interestingly, we also uncovered a 7-gene eCAG (Pro-eCAG_006 and Syn-eCAG_002), encompassing a putative nitrilase gene (*nitC*), which also suggests that most *Synechococcus* cells and a more variable fraction of the *Prochlorococcus* population could use nitriles or cyanides in warm, Fe-replete waters and more particularly in low-N areas such as the Indian Ocean (Fig. 5A, B). The whole operon (*nitHBCDEFG;* Fig. 5C), called Nit1C, was shown to be upregulated in the presence of cyanide and to trigger an increase in the rate of ammonia accumulation in the heterotrophic bacterium *Pseudomonas fluorescens* [49], suggesting that like cyanate, cyanide could constitute an alternative nitrogen source in marine picocyanobacteria as well. However, given the potential toxicity of these C ≡ N-containing compounds [50], we cannot exclude that these eCAGs could also be devoted to cell detoxification [45, 47]. Such an example of detoxification has been described for arsenate and chromate that, as analogs of phosphate and sulfate respectively, are toxic to marine phytoplankton and must be actively exported out of the cells [51, 52].

We detected the presence of an eCAG encompassing *asnB, pyrB2*, and *pydC* (Pro-eCAG_007, Syn-eCAG_003, Fig. S8), which could contribute to an alternative pyrimidine biosynthesis pathway and thus provide another way for cells to recycle complex nitrogen forms. While this eCAG is found in only one fifth of HLII genomes and in quite specific locations for *Prochlorococcus*, notably in the Red Sea, it is found in most *Synechococcus* cells in warm, Fe-replete, N and P-depleted niches, consistent with its phyletic pattern showing its absence only from most clade I, IV, CRD1, and EnvB genomes (Fig. S8; Dataset 6). More generally, most N-uptake and assimilation genes in both genera were specifically absent from Fe-depleted areas, including the *nirA/narB* eCAG for *Prochlorococcus*, as mentioned by Kent et al. [36] as well as guanidinase and nitrilase eCAGs. In contrast, picocyanobacterial populations present in low-Fe areas possess, in addition to the core ammonium transporter *amt1*, a second transporter *amt2*, also present in cold areas for *Synechococcus* (Fig. S9). Additionally, *Prochlorococcus* populations thriving in HNLC areas also possess two amino acid-related eCAGs that are present in most *Synechococcus* genomes, the first one involved in polar amino acid N-II transport (Pro-eCAG_008; *natF-G-H-bgtA;* [53]; Fig. S10A, B) and the second one (*leuDH-soxA-*CK_00001744, Pro-eCAG_009, Fig. S10C, D) that notably encompasses a leucine dehydrogenase, able to produce ammonium from branched-chain amino acids. This highlights the profound difference in N acquisition mechanisms between HNLC regions and Fe-replete, N-deprived areas: the primary nitrogen sources for picocyanobacterial populations dwelling in HNLC areas seem to be ammonium and amino acids, while N acquisition mechanisms are more diverse in N-limited, Fe-replete regions.

#### eCAGs related to phosphorus metabolism

Adaptation to P depletion has been well documented in marine picocyanobacteria showing that while in P-replete waters *Prochlorococcus* and *Synechococcus* essentially rely on inorganic phosphate acquired by core transporters (PstSABC), strains isolated from low-P regions and natural populations thriving in these areas additionally contain a number of accessory genes related to P metabolism, located in specific genomic islands [6, 14, 30–32, 54]. Here, we indeed found in *Prochlorococcus* an eCAG containing the *phoBR* operon (Pro-eCAG_010) that encodes a two-component system response regulator, as well as an eCAG including the alkaline phosphatase *phoA* (Pro-eCAG_011), both present in virtually the whole *Prochlorococcus* population from the Mediterranean Sea, the Gulf of Mexico and the Western North Atlantic Ocean, which are known to be P-limited [30, 55] (Fig. S11A, B). By comparison, in *Synechococcus*, we only identified the *phoBR* eCAG (Syn-eCAG_005, Fig. S11C) that is systematically present in warm waters whatever the limiting nutrient, in agreement with its phyletic pattern in reference genomes showing its specific absence from cold thermotypes (clades I and IV, Dataset 6). Furthermore, although our analysis did not retrieve them within eCAGs due to the variability of gene content and synteny in this genomic region, even within each genus, several other P-related genes were enriched in low-P areas but partially differed between *Prochlorococcus* and *Synechococcus* (Figs. 3, S2, S11; Dataset 6). While the genes putatively encoding a chromate transporter (ChrA) and an arsenate efflux pump ArsB were present in both genera in different proportions, a putative transcriptional phosphate regulator related to PtrA (CK_00056804; [56]) was specific to *Prochlorococcus*. *Synechococcus* in contrast harbors a large variety of alkaline phosphatases (PhoX, CK_00005263 and CK_00040198) as well as the phosphate transporter SphX (Fig. S11).

**Fig. 6:**
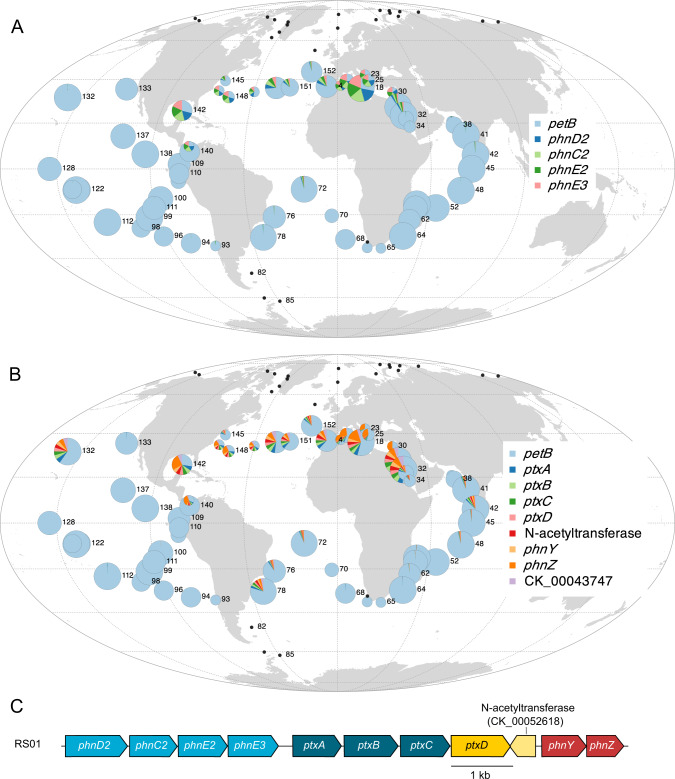
Global distribution map of eCAGs putatively involved in phosphonate and phosphite transport and assimilation. **A** *Prochlorococcus* Pro-eCAG_012 putatively involved in phosphonate transport. **B** *Prochlorococcus* Pro-eCAG_013, involved in phosphonate/phosphite uptake and assimilation and phosphonate C-P bond cleavage. **C** The genomic region encompassing both *phnC2-D2-E2-E3* and *ptxABDC-phnYZ* specific to *P. marinus* RS01. The size of the circle is proportional to relative abundance of *Prochlorococcus* as estimated based on the single-copy core gene *petB* and this gene was also used to estimate the relative abundance of other genes in the population. Black dots represent *Tara* Oceans stations for which *Prochlorococcus* read abundance was too low to reach the threshold limit.

Phosphonates, i.e. reduced organophosphorus compounds containing C–P bonds that represent up to 25% of the high-molecular-weight dissolved organic P pool in the open ocean, constitute an alternative P form for marine picocyanobacteria [57]. We indeed identified, in addition to the core phosphonate ABC transporter (*phnD1-C1-E1*), a second previously unreported putative phosphonate transporter *phnC2-D2-E2-E3* (Pro-eCAG_012; Fig. 6A). Most of the *Prochlorococcus* population in strongly P-limited areas of the ocean harbored these genes, while they were absent from other areas, consistent with their presence in only a few *Prochlorococcus* and no *Synechococcus* genomes. Furthermore, as previously described [58–60], we found a *Prochlorococcus* eCAG encompassing the *phnYZ* operon involved in C-P bond cleavage, the putative phosphite dehydrogenase *ptxD*, and the phosphite and methylphosphonate transporter *ptxABC* (Pro-eCAG_0013, Dataset 6; Fig. 6B, [60–62]). Compared to these previous studies that mainly reported the presence of these genes in *Prochlorococcus* cells from the North Atlantic Ocean, here we show that they actually occur in a much larger geographic area, including the Mediterranean Sea, the Gulf of Mexico, and the ALOHA station (TARA_132) in the North Pacific, even though they were present in a fairly low fraction of *Prochlorococcus* cells. These genes occurred in an even larger proportion of the *Synechococcus* population, although not found in an eCAG for this genus (Fig. S12; Dataset 6). *Synechococcus* cells from the Mediterranean Sea, a P-limited area dominated by clade III [24], seem to lack *phnYZ*, in agreement with the phyletic pattern of these genes in reference genomes, showing the absence of this two-gene operon in the sole clade III strain that possesses the *ptxABDC* gene cluster. In contrast, the presence of the complete gene set (*ptxABDC-phnYZ*) in the North Atlantic, at the entrance of the Mediterranean Sea, and in several clade II reference genomes rather suggests that it is primarily attributable to this clade. Altogether, our data indicate that part of the natural populations of both *Prochlorococcus* and *Synechococcus* would be able to assimilate phosphonate and phosphite as alternative P-sources in low-P areas using the *ptxABDC-phnYZ* operon. Yet, the fact that no picocyanobacterial genome except *P. marinus* RS01 (Fig. 6C) possesses both *phnC2-D2-E2-E3* and *phnYZ*, suggests that the phosphonate taken up by the *phnC2-D2-E2-E3* transporter could be incorporated into cell surface phosphonoglycoproteins that may act to mitigate cell mortality by grazing and viral lysis, as recently suggested [63].

#### eCAGs related to iron metabolism

As for macronutrients, it has been hypothesized that the survival of marine picocyanobacteria in low-Fe regions was made possible through several strategies, including the loss of genes encoding proteins that contain Fe as a cofactor, the replacement of Fe by another metal cofactor, and the acquisition of genes involved in Fe uptake and storage [14, 15, 36, 39, 64]. Accordingly, several eCAGs encompassing genes encoding proteins interacting with Fe were found in modules anti-correlated to HNLC regions in both genera. These include three subunits of the (photo)respiratory complex succinate dehydrogenase (SdhABC, Pro-eCAG_014, Syn-eCAG_006, Fig. S13; [65]) and Fe-containing proteins encoded in most abovementioned eCAGs involved in N or P metabolism, such as the guanidinase (Fig. S6), the NitC1 (Fig. 5), the *pyrB2* (Fig. S8), the phosphonate (Fig. 6, S12), and the urea and inorganic nitrogen eCAGs (Fig. S5). Most *Synechococcus* cells thriving in Fe-replete areas also possess the *sodT*/*sodX* eCAG (Syn-eCAG_007, Fig. S14A, B) involved in nickel transport and maturation of the Ni-superoxide dismutase (SodN), these three genes being in contrast core in *Prochlorococcus*. Additionally, *Synechococcus* from Fe-replete areas, notably from the Mediterranean Sea and the Indian Ocean, specifically possess two eCAGs (Syn-eCAG_008 and 009; Fig. S14C, D), involved in the biosynthesis of a polysaccharide capsule that appear to be most similar to the *E. coli* groups 2 and 3 *kps* loci [66]. These extracellular structures, known to provide protection against biotic or abiotic stress, were recently shown in *Klebsiella* to provide a clear fitness advantage in nutrient-poor conditions since they were associated with increased growth rates and population yields [67]. However, while these authors suggested that capsules may play a role in Fe uptake, the significant reduction in the relative abundance of *kps* genes in low-Fe compared to Fe-replete areas (*t*-test *p*-value < 0.05 for all genes of the Syn-eCAG_008 and 009; Fig. S14C) and their absence in CRD1 strains (Dataset 6) rather suggests that these capsules may be too energy-consuming for some picocyanobacteria thriving in this particular niche, while they may have a more meaningful and previously overlooked role in their adaptation to low-P and low-N niches.

**Fig. 7:**
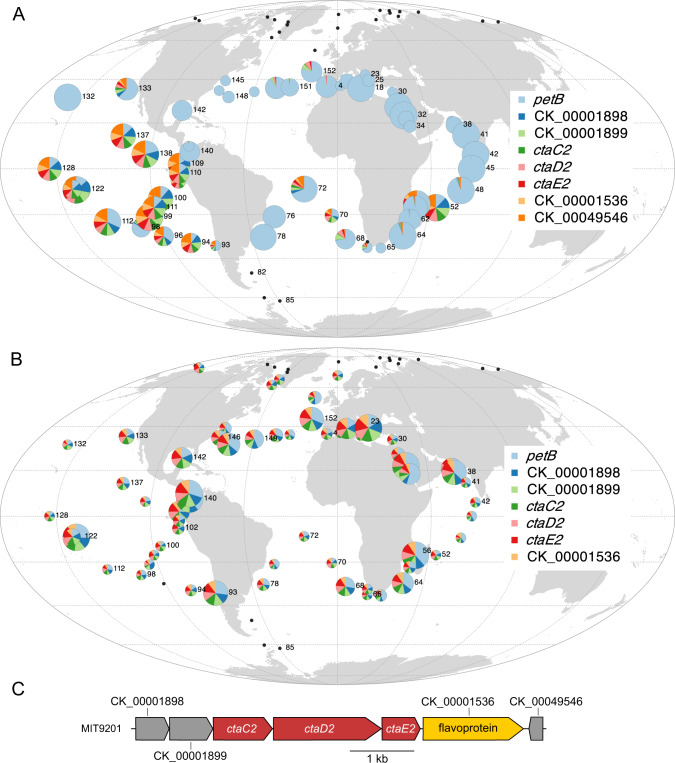
Global distribution map of the *Prochlorococcus* eCAGs involved in the biosynthesis of an alternative respiratory terminal oxidase (ARTO). **A** *Prochlorococcus* Pro-eCAG_016. **B** *Synechococcus* Syn-eCAG_015. **C** The genomic region encompassing the *ctaC2D2E2* operon in *P. marinus* MIT9201. The size of the circle is proportional to relative abundance of each genus as estimated based on the single-copy core gene *petB* and this gene was also used to estimate the relative abundance of other genes in the population. Black dots represent *Tara* Oceans stations for which *Prochlorococcus* or *Synechococcus* read abundance was too low to reach the threshold limit.

Several eCAGs were in contrast enriched in populations dwelling in HNLC environments, dominated by *Prochlorococcus* HLIIIA/HLIVA/LLIB and *Synechococcus* CRD1A/EnvBA ESTUs (Fig. 2). The vast majority of *Prochlorococcus* cells thriving in low-Fe regions possess an eCAG encompassing the *ctaC2-D2-E2* operon, also found in 85% of all *Synechococcus* reference genomes, including all CRD1 (Fig. 7; Dataset 6). This eCAG encodes the alternative respiratory terminal oxidase ARTO, a protein complex that has been suggested to be part of a minimal respiratory chain in the cytoplasmic membrane, potentially providing an additional electron sink under Fe-deprived conditions [68, 69]. Furthermore, a *Synechocystis* mutant in which the *ctaD2* and *ctaE2* genes were inactivated was found to display markedly impaired Fe reduction and uptake rates as compared to wild-type cells, suggesting that ARTO is involved in the reduction of Fe(III) to Fe(II) prior to its transport through the plasma membrane via the Fe(II) transporter FeoB [70]. Thus, the presence of the ARTO system appears to represent a major and previously unreported adaptation for *Prochlorococcus* populations thriving in low-Fe areas.

Both *Prochlorococcus* and *Synechococcus* thriving in low-Fe waters also possess eCAGs encoding the TonB-dependent siderophore uptake operon *fecDCAB-tonB*-*exbBD* (Pro-eCAG_015 and Syn-eCAG_013-014, Dataset 6). This gene cluster, which is found in a few picocyanobacterial genomes and was previously shown to be anti-correlated with dissolved Fe concentration [14, 15, 64], is indeed systematically present in a significant part of the *Prochlorococcus* and *Synechococcus* population in low-Fe areas (Fig. S15). However, it is also present in a small fraction of the populations thriving in the Indian Ocean, consistent with its occurrence in two *Prochlorococcus* HLII and one *Synechococcus* clade II genomes (Dataset 6). Finally, a large proportion of the *Synechococcus* populations in HNLC areas possess (i) a large eCAG involved in glycine betaine synthesis and transport (Syn-eCAG_010, Fig. S15A, B; [6, 71, 72]), almost absent from low-N areas, (ii) an eCAG encompassing a flavodoxin and a thioredoxin reductase (Syn-eCAG_011, Fig. S16C, D), mostly absent from low-P areas, and (iii) the *nfeD-floT1-floT2* eCAG (Syn-eCAG_012, Fig. S17A, B) involved in the production of lipid rafts, potentially affecting cell shape and motility [6, 73].

#### eCAGs enriched in cold waters

Besides genes involved in nutrient acquisition and metabolism, several *Prochlorococcus* and *Synechococcus* eCAGs were found to be correlated with low temperature. A closer examination of *Prochlorococcus* eCAGs however, shows that their occurrence is not directly related to temperature adaptation but mainly explained by the prevalence at high latitude of either (i) the HLIA ESTU (Figs. 2A, C, 4), the *red* module encompassing most of the above-mentioned eCAGs involved in P-uptake and assimilation pathways, or (ii) the LLIA ESTU, present in surface waters at vertically-mixed stations, the *turquoise* module mainly gathering *Prochlorococcus* LL-specific genes, such as Pro-eCAG_017, involved in phycoerythrin-III biosynthesis (*ppeA*, *cpeFTZY*, *unk13*). As concerns *Synechococcus*, although a fairly high number of eCAGs were identified in the *tan* module associated with ESTUs IA and IVA-C (Fig. 2B, D and Fig. S4), only very few are conserved in more than two reference strains and/or have a characterized function (Dataset 6). Among these, at least one eCAG is clearly related to adaptation to cold waters, the orange carotenoid protein (OCP) operon (*ocp*-*crtW*-*frp*; Syn-eCAG_016). Indeed, this operon is involved in a photoprotective process [74] that provides cells with the ability to deal with oxidative stress under cold temperatures [75]. Accordingly, our data shows that *Synechococcus* populations colonizing mixed waters at high latitudes or in upwelling areas all possess this eCAG (Fig. S18), highlighting the importance of this photoprotection system in *Synechococcus* populations colonizing cold and temperate areas. *Synechococcus* populations thriving in cold waters also appear to be enriched in eCAGs involved in gene regulation such as transcription factors involved in the regulation of the CA4-A form of the type IV chromatic acclimation process (*fciA-B;* Syn-eCAG_017), consistent with the predominance of *Synechococcus* CA4-A cells in temperate or cold environments [76–78] (Dataset 6). Altogether, the fairly low number of eCAGs strongly associated with temperature supports the hypothesis that adaptation to cold temperature is not mediated by evolution of gene content but rather of protein sequences [5, 6, 36, 79].

## Conclusions

Our analysis of *Prochlorococcus* and *Synechococcus* gene distributions at the global scale using the deeply sequenced metagenomes collected along the *Tara* Oceans expedition transect revealed that each picocyanobacterial community has a specific gene repertoire, with different sets of accessory genes being highly correlated with distinct ESTUs and physicochemical parameters. As previously suggested for *Prochlorococcus* [36], this strong correlation between taxonomy and gene content strengthens the idea that, in both genera, genome evolution mainly occurs by vertical transmission and selective gene retention, and that lateral gene transfers between ecotypes are fairly scarce. By combining information about gene synteny in 256 reference genomes with the distribution and abundance of these genes in the field, we further managed to delineate suites of adjacent genes likely involved in the same metabolic pathways that may have a crucial role in adaptation to specific niches. These analyses confirmed previous observations about the niche partitioning of individual genes and a few genomic regions involved in nutrient uptake and assimilation [14, 15, 25, 31, 33, 36, 40, 42]. Most importantly, even though our network approach likely only revealed the lower boundary of the number of eCAGs actually present in different niches, due to the incompleteness of some reference genomes, this approach highlighted that some previously detected individual genes are part of larger genomic regions and unveiled several novel genomic regions. Although we cannot exclude that some genes enriched in a specific niche are not adaptive per se but either hitchhiked along with an adaptive gene [80] or occurred from passive transport of ecotype populations outside their niche [79, 81], it is reasonable to assume that many eCAGs identified in the present study could confer cells a fitness benefit in particular niches and were thus retained by natural selection, or in contrast have been counter-selected in certain environments (such as eCAGs that are absent from low-Fe environments). This study revealed the potential importance of the uptake and assimilation of organic forms of nutrients, which might either be directly used by cells e.g. for the biosynthesis of proteins or DNA, or be degraded into inorganic N and/or P forms. Consequently, many eCAGs potentially involved in the uptake and assimilation of complex compounds, such as guanidine, C ≡ N-containing compounds, or pyrimidine were present in both N- and P-depleted waters, and might constitute an advantage in areas of the world ocean co-limited in these nutrients [30], while they were absent from HNLC areas. Our data also suggests that adaptation to Fe-limitation relies on specific adaptation mechanisms including the reduction of Fe(III) to Fe(II) using ARTO, Fe scavenging using siderophores, as well as reduction of the Fe quota and of energy-consuming mechanisms, such as polysaccharide capsule biosynthesis. Altogether, this study provides novel insights into the genetic basis of niche partitioning in key members of the phytoplankton community. A future challenge will clearly be biochemically characterizing the function of adaptive genes in these eCAGs (Datasets 5, 6), which are sometimes present in only a few or even a single cultured strain but which can occur in a large part or even the whole *Prochlorococcus* and/or *Synechococcus* population occupying a specific niche in situ.

## Materials and methods

*Tara* Oceans metagenomic reads from surface waters corresponding to the bacterial size-fraction [24, 82] were recruited against 256 reference genomes using MMseqs2 Release 11-e1a1c (76) and then mapped to an extended database, including 722 outgroup cyanobacterial genomes (Dataset 9; Supplementary Methods). Picocyanobacterial reads were then taxonomically assigned to either *Prochlorococcus* or *Synechococcus* and functionally assigned to a cluster of likely orthologous genes (CLOGs) as defined in the information system Cyanorak *v2.1* [19]. After normalization by gene and read length, filtration steps included the selection of (i) samples containing more CLOGs than the average number of genes in a *Synechococcus* or *Prochlorococcus* HL genome, (ii) CLOGs with a gene coverage higher than 1 in at least 2 of the selected samples and (iii) non-core genes [6].

*Tara* Oceans stations were clustered using Ward’s minimum variance [83] based on Bray-Curtis similarities between the relative abundance of either CLOG or picocyanobacterial ESTUs as defined based on the *petB* marker gene [24]. CLOG abundance profiles were also used to perform co-occurrence analyses by weighted genes correlation network analysis (WGCNA, [84, 85]) to delineate modules of CLOGs that share a similar distribution pattern. The *eigengene* of each module was then correlated to environmental parameters, retrieved from PANGAEA (www.pangaea.de/), and to the relative abundance of *petB* ESTUs. Furthermore, the most representative genes of each module were identified as those most correlated to the *eigengene*.

Finally, we then defined eCAGs within each module by searching adjacent genes (less than 6 genes apart in 80% of the genomes possessing them) in the 256 reference genomes (Datasets 7, 8) that were used to build a network of the corresponding CLOGs (node) according to the graph embedder (GEM) or the Fruchterman-Reingold layout algorithms implemented in the R package igraph [86].

## Supplementary information

Dataset 1

Dataset 2

Dataset 3

Dataset 4

Dataset 5

Dataset 6

Dataset 7

Dataset 8

Dataset 9

Supplementary information

## Data Availability

All genome sequences used in this study are available from NCBI as detailed in Dataset 9, while *Tara* Oceans metagenomes and corresponding environmental parameters were retrieved from PANGAEA (www.pangaea.de/).
