# Supplementary material for: Differential global distribution of marine picocyanobacteria gene clusters reveals distinct niche-related adaptive strategies
Source: ISME J. 2023 Feb 25;17(5):720–32. doi: 10.1038/s41396-023-01386-0 (PMC10119275; doi:10.1038/s41396-023-01386-0)
Supplement: Supplementary file 10 — Supplementary information [file 41396_2023_1386_MOESM10_ESM.pdf]

Supplementary Information for

Differential global distribution of marine picocyanobacteria gene clusters reveals distinct niche-related adaptive strategies

Hugo Doré[a,1], Ulysse Guyet [a,1], Jade Leconte[a], Gregory K. Farrant[a], Benjamin Alric[a], Morgane Ratin[a], Martin Ostrowski[b,2], Mathilde Ferrieux[a], Loraine Brillet-Guéguen[c,d], Mark Hoebeke[c], Jukka Siltanen[c], Gildas Le Corguillé[c], Erwan Corre[c], Patrick Wincker[f,g], David J. Scanlan[b], Damien Eveillard[h,g], Frédéric Partensky[a], and Laurence Garczarek[a,g,3]

Email: laurence.garczarek@sb-roscoff.fr

This PDF file includes:

Supplementary methods
Supplementary text
Figures S1 to S18
SI References

Supplementary methods

Tara Oceans dataset

A total of 131 bacterial-size metagenomes (0.2-1.6 µm for stations TARA_004 to TARA_052 and 0.2-3µm for TARA_056 to TARA_152), collected in surface waters from 83 stations along the *Tara* Oceans expedition transect [1], were used in this study. Briefly, all metagenomes were sequenced as Illumina overlapping paired reads of 100-108 bp and paired reads were merged and trimmed based on quality, resulting in 100-215 bp fragments, as previously described [2]. All metagenomes and corresponding environmental parameters were retrieved from PANGAEA (http://www.pangaea.de/) except for Fe and ammonium concentrations that were modeled and the Fe limitation index Φ_{sat} that was calculated from satellite data, as previously described [2].

Recruitment and taxonomic and functional assignment of metagenomic reads

Metagenomic reads were first recruited against 256 reference genomes, including the 97 genomes available in the information system Cyanorak *v2.1* (www.sb-roscoff.fr/cyanorak; (28)) as well as 84 additional WGS, 27 MAGs and 48 SAGs retrieved from Genbank (Dataset 9). Recruitment was made using MMseqs2 Release 11-e1a1c (76) with maximum sensitivity (mmseqs search -s 7.5) and limiting the results to one target sequence (mmseqs filterdb --extract-lines 1). Using the same MMseqs2 options, the resulting reads were then mapped to an extended database of 978 genomes, including all picocyanobacterial reference genomes complemented with 722 outgroup cyanobacterial genomes downloaded from NCBI. Reads mapping to outgroup sequences or having less than 90% of their sequence aligned were filtered out and the remaining reads were taxonomically assigned to either *Prochlorococcus* or *Synechococcus* according to their best hit. Reads were then functionally assigned to a cluster of likely orthologous genes (CLOGs) from the information system Cyanorak *v2.1* based on the position of their MMseqs2 match on the genome, the coordinates of which correspond to a particular gene in the database. More precisely, a read was functionally assigned to a gene if at least 75% of its size was aligned to the reading frame of this gene and if the percentage identity of the blast alignment was over 80%. Finally, read counts were aggregated by CLOG and normalized by dividing read counts by L-l+1, where L represents the average gene length of the CLOG and l the mean length of recruited reads. Only environmental samples that contained at least 2,500 and 1,700 distinct CLOGs for *Synechococcus* and *Prochlorococcus,* respectively, were kept, corresponding roughly to the average number of genes in a *Synechococcus* and a *Prochlorococcus* HL genome, respectively. After this filtration step, a CLOG was kept if it showed a gene-length normalized abundance higher than 1 (i.e., a gene coverage of 1) in at least 2 of the selected environmental samples. Then, large-core genes, as previously defined [3], were removed to keep only accessory genes. The resulting abundance profiles were used to perform co-occurrence analyses by weighted genes correlation network analysis, as detailed below (WGCNA, [4]).

Station clustering and ESTU analyses

In order to cluster stations displaying similar CLOG abundance patterns, the abundance of a given CLOG in a sample was divided by the total CLOG abundance in this sample to obtain relative abundance profiles per sample. Bray-Curtis similarities were calculated from these profiles and used to cluster *Tara* Oceans stations with the Ward's minimum variance method [5]. The same normalization method was applied to picocyanobacterial ESTUs that were defined based on the *petB* marker gene at each station using a similar approach as in Farrant *et al.* (2016) but using a Ward's minimum variance method [5] to be consistent with the clustering of CLOG profiles. In order to check whether the Bray-Curtis distances between stations based on *petB* picocyanobacterial communities and based on gene content were significantly correlated, a mantel test was performed between the distance matrices, as implemented in the R package *vegan* v2.5 with 9,999 permutations [6].

Gene co-occurrence network analysis

A data-reduction approach based on WGCNA, as implemented in the R package WGCNA *v1.51* [7], was used to build a co-occurrence network of CLOGs based on their relative abundance in *Tara* Oceans stations and to delineate modules of CLOGs (i.e., subnetworks). The WGCNA

adjacency matrix was calculated in 'signed' mode (i.e., considering correlated and anti-correlated CLOGs separately), by using the *Pearson* correlation between pairs of CLOGs (based on their relative abundance in every sample) and raising it to the power 12, which allowed to obtain a scale-free topology of the network. Modules were identified by setting the minimum number of genes in each module to 100 and 50 for *Synechococcus* and *Prochlorococcus,* respectively, and by forcing every gene to be included in a module. The *eigengene* of each module, i.e. the first principal component of gene abundances at the different stations for this module (representative of the relative abundance of genes of a given module at each *Tara* Oceans station) was then correlated to environmental parameters and to the relative abundance of ESTUs. Finally, genes in each module with the highest correlation to the *eigengene* (a measurement called 'membership'), were extracted in order to identify the most representative genes of each module.

Identification of differentially distributed clusters of adjacent genes (eCAGs)

Results on individual niche-related genes identified by WGCNA were then integrated with knowledge on gene synteny in reference genomes (Datasets 7 and 8). For each WGCNA module, we defined eCAGs by searching adjacent genes of the module in the 256 reference genomes. In order to be considered as belonging to the same eCAG, two genes of the same module must be less than 6 genes apart in 80% of the genomes in which these two genes are present. The 80% cut-off allowed us to take into account the incompleteness of some reference genomes, and notably MAGs and SAGs. This method led us to identify clusters of adjacent genes in reference genomes, comprising genes displaying a similar distribution pattern, called eCAGs. It is worth noting that the association of an eCAG with a specific niche is totally independent from gene synteny. Indeed, this association is based on the fact that all genes in an eCAG necessarily come from the same WGCNA module, which is itself associated with a niche. Thus, the potential absence of whole syntenic genome regions in MAGs due to assembly biases cannot lead to false associations of an eCAG with a particular environment. Furthermore, none of the eCAGs mentioned in the text (Dataset 6) were exclusively present in MAGs, excluding the risk of false positives due to MAG assembly biases.

A network of eCAGs was then built for each WGCNA module, considering the number of genomes in which these genes are adjacent (Figs. 4, S3 and S4). A graph representation of eCAGs was displayed using the graph embedder (GEM) algorithm [8] or the Fruchterman-Reingold algorithm [9] implemented in the R-package igraph [10]. These are force-directed algorithms, meaning that node layout is determined by the forces pulling nodes together and pushing them apart. In other words, its purpose is to position the nodes of a graph so that the edges of more or less equal length are gathered together and to avoid as many crossing edges as possible. The first algorithm was used to draw an unweighted and undirected global graph of all eCAGS (Fig. 4). The second algorithm was used to draw, for each WGCNA module separately, unweighted and undirected graphs of eCAGS where link thickness corresponds to the number of genomes in which the eCAG members are less than six genes apart (Figs. S3, S4).

Supplementary Information Text

Description of picocyanobacterial WGCNA modules and correlations with environmental parameters and ESTUs.

***Prochlorococcus* modules**

In order to better interpret the global distribution of picocyanobacterial gene content, gene modules obtained by WGCNA were correlated to the available environmental parameters (Figs. 2A-B, S1A-B) and the relative abundance of *Prochlorococcus* or *Synechococcus* ESTUs at each station (Fig. 2C-D, S1A-B). The brown module, corresponding to genes preferentially found in Fe-limited HNLC areas and strongly associated with the presence of HLIIIA, HLIVA and LLIB, is described in the main text. The blue module was found to be associated with warm, low-chlorophyll oligotrophic regions with low N and P concentrations and high Fe availability (Fig. 2A), where ESTUs HLIIA dominate the *Prochlorococcus* community and HLIIB-D were also present at lower abundance (Fig. 2C, Fig. S1A). The turquoise module seems to correspond to genes present in cold, chlorophyll-rich waters, colonized by LLIA ESTUs, and to a lower extent to LLIC and LLID, but anti-correlated with HLII ESTUs. The turquoise module gathers station TARA-070, where LLIA dominates (Fig. 1A), as well as stations dominated either by HLIA or the coldest stations dominated by HLIIA ESTUs (TARA-0146 and 149), the common point between all these stations being a strong relative abundance of LLIA (Figs. 1 and S1A). Finally, the red module seems to be characteristic of cold, Fe-rich, N- and P-depleted waters, and strongly correlated to HLIA and anti-correlated to HLII-IV and LLIB ESTUs (Fig. 2A-C), corresponding to assemblages mainly found at the highest latitude stations of the *Tara* Oceans transect (TARA_066, 068, 093, 094, 133, 150, 151, 152) as well as all stations in the Mediterranean Sea (Fig. S1A).

***Synechococcus* modules**

The same analysis performed on *Synechococcus* genes shows that the yellow module is correlated with phosphate and ammonium concentrations and strongly anti-correlated to Fe availability, and thus corresponds to genes found in HNLC areas. Accordingly, this module is correlated to ESTUs CRD1A, CRD1C, EnvAA and EnvBA, previously reported to dwell in Fe-depleted areas ([2, 8]; Figs. 2B-D). Although the midnight blue module is only positively correlated with oxygen concentration, it is most strongly associated with ESTU IA and IVA-C, known to colonize cold, coastal, or mixed open ocean waters at high latitude [2] and anti-correlated with ESTUs IIA and IIIA/B (Fig. 2C-D). In terms of distribution, genes of this module are only found in two upwelling stations (TARA_093, 133), as well as in a cold station sampled in winter at the northernmost Atlantic station of the *Tara* Ocean transect, TARA_152 (Figs. 1B, S1B in this study and Fig.4 in [2]). The tan module was found in cold, chlorophyll-rich waters with a high relative abundance of ESTUs IA and IVA-C (Fig. 2B-D) and was also detected in the most strongly mixed waters of the *Tara* Oceans dataset, notably the upwelling stations (TARA_067, 093), at TARA_145, a cold station sampled in winter, North of the Gulf stream as well as in northern Atlantic stations of the *Tara* Ocean transect (TARA_150, 151, 152, Fig. S1B). The purple module is found in waters with high salinity, iron-rich, P-depleted waters, and associated with IIIA/B, WPC1A and all SC 5.3 ESTUs, known to co-occur in low-P areas of the world ocean (Fig. 2B-D). Consistently, it was specifically found in the Mediterranean Sea and the only station of the Gulf of Mexico (TARA_142, Fig. S1B). Finally, the salmon module was associated with warm, Fe-rich waters. This module was most strongly associated with ESTU IIA and to a lesser extent with the fairly rare ESTUs VIIA and 5.3B and its eigengene accordingly has higher values at stations dominated by ESTU IIA (Fig. S1).

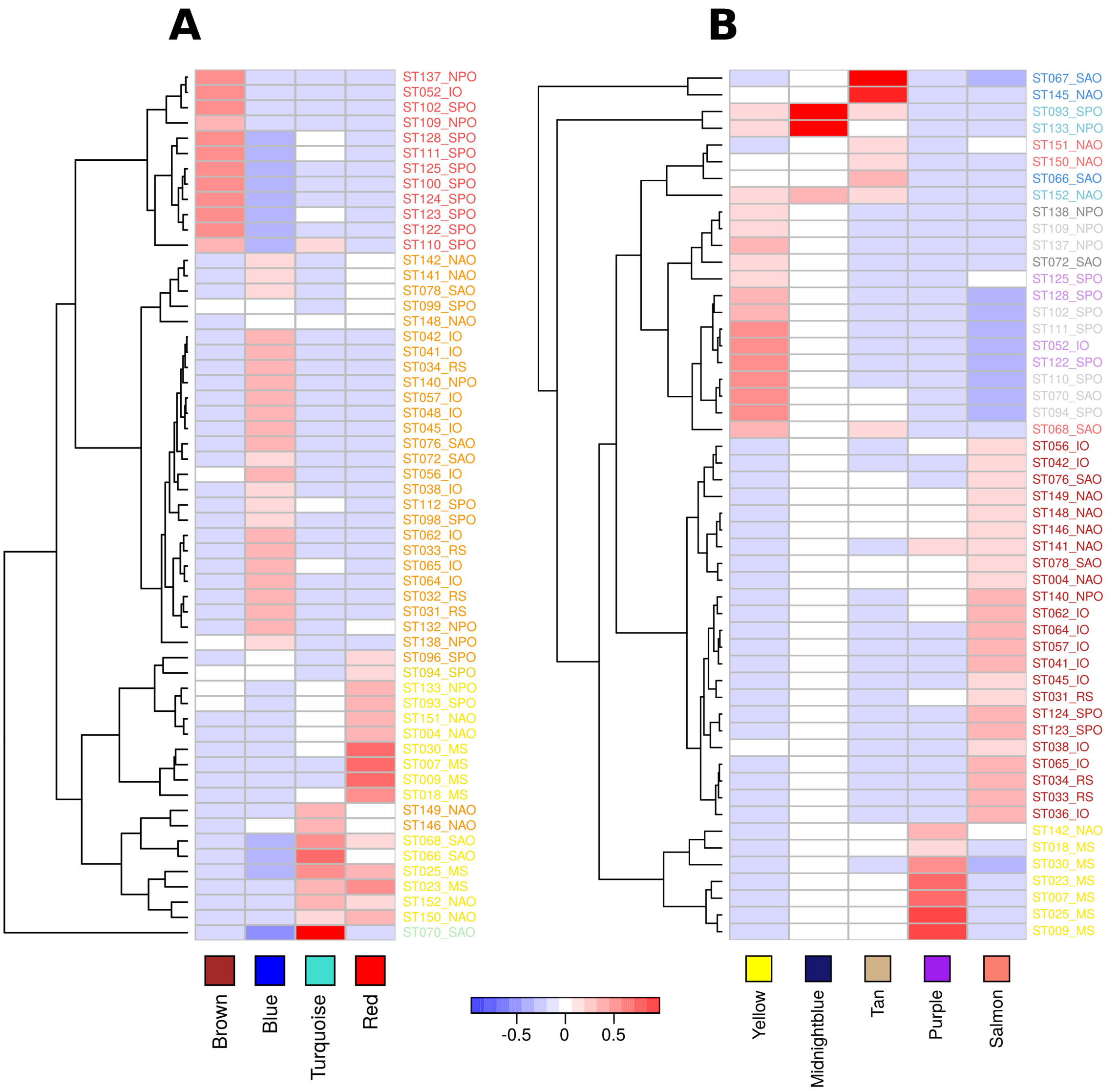

Fig. S1. Distribution of the eigengene of each WGCNA module. *Prochlorococcus* (*A*) and *Synechococcus* (*B*) modules are designated by color names indicated below each heatmap. The *eigengene* of a given module represents a consensus of the normalized relative abundance of genes of that module in *Tara* Oceans stations. Station names are colored according to ESTU assemblages defined in Farrant et al. (2016) and specify the oceanic region of each station as follows: SAO, South Atlantic Ocean; MS, Mediterranean Sea; NAO, North Atlantic Ocean; IO, Indian Ocean; RS, Red Sea; SPO, South Pacific Ocean; NPO, North Pacific Ocean.

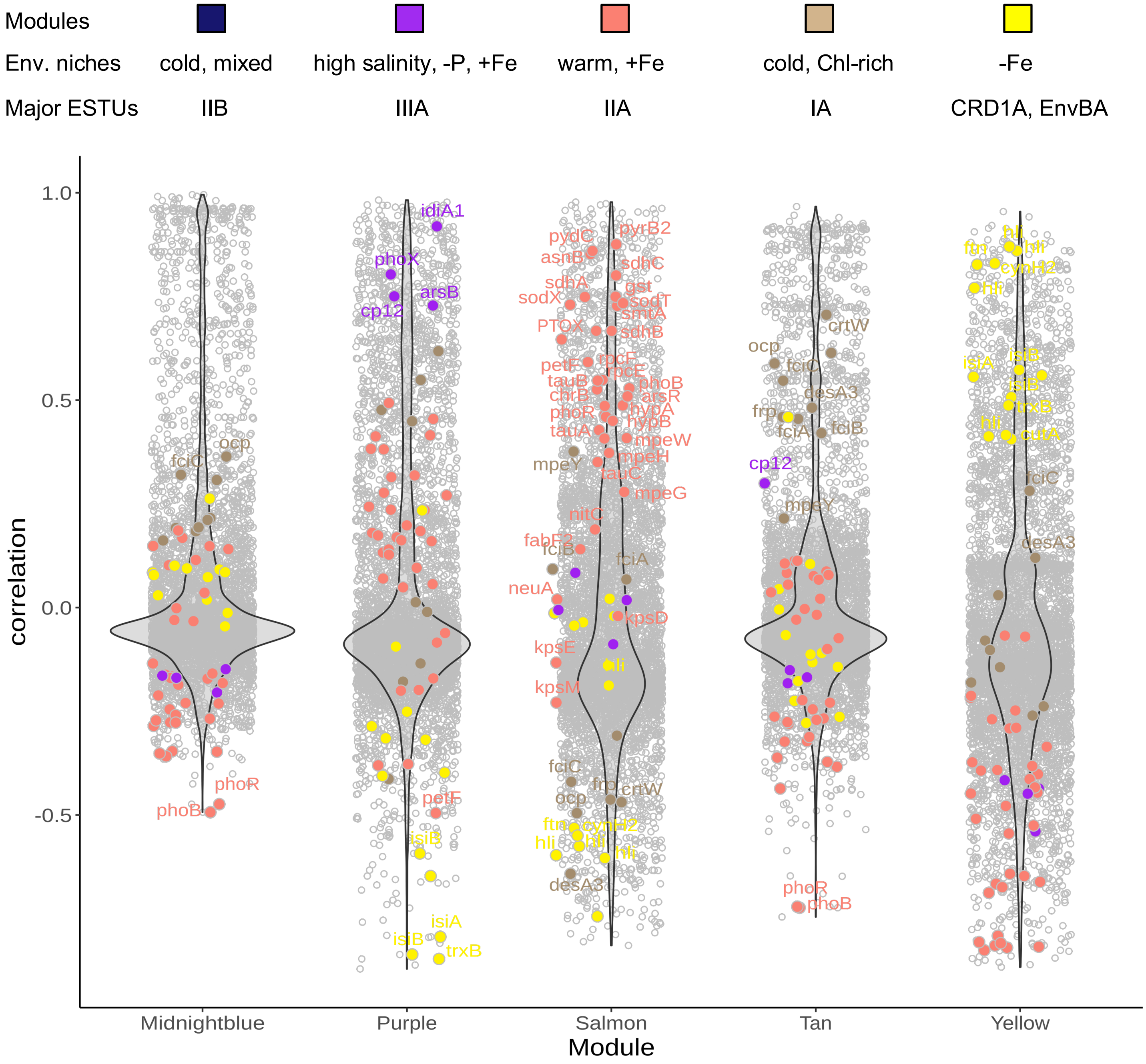

Fig. S2. Same as Fig. 3 for *Synechococcus*

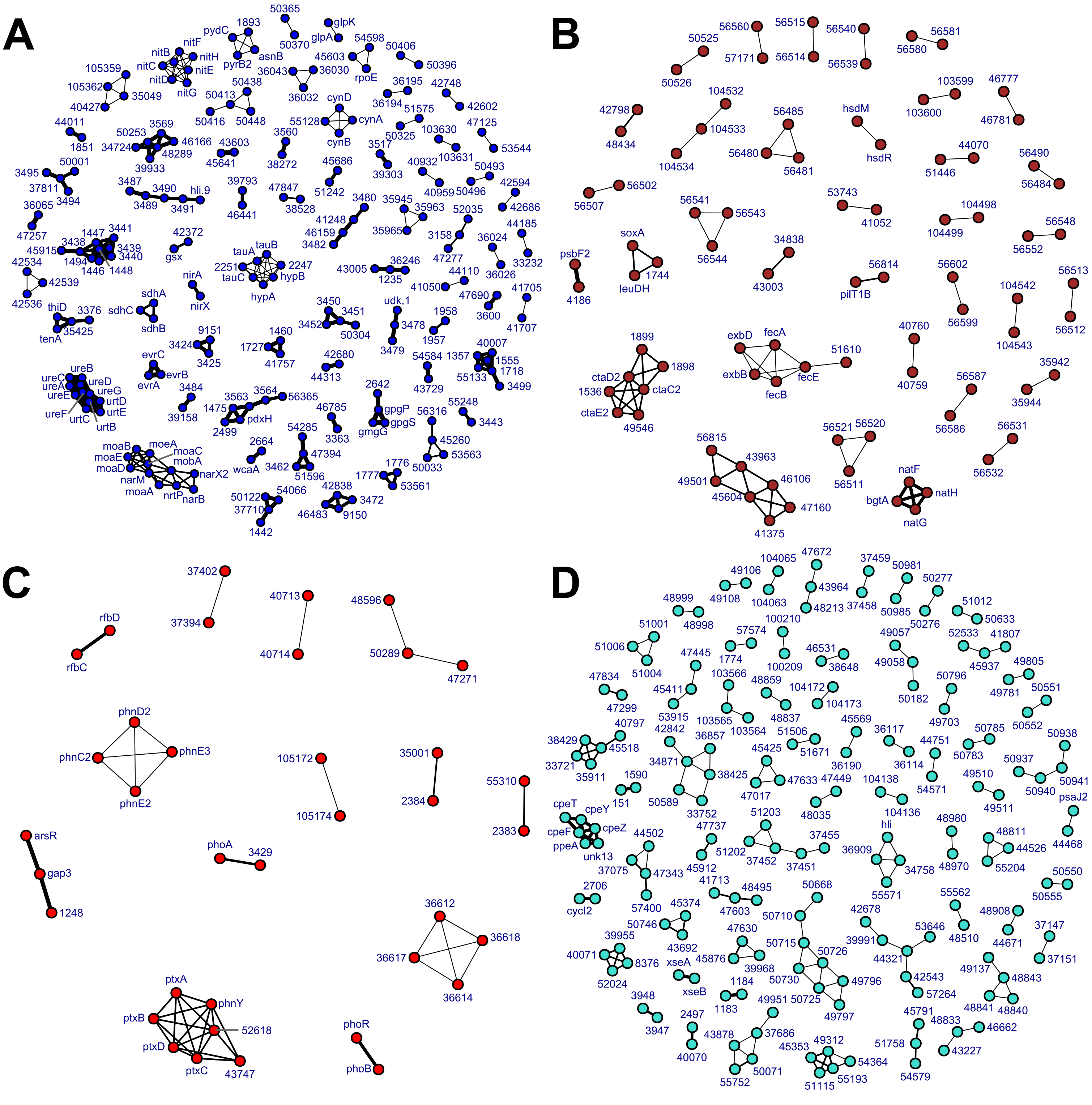

Fig. S3. Same as Fig. 4 but for each individual *Prochlorococcus* WGCNA module. A link between two nodes indicates that these two genes are less than 6 genes apart in at least one genome and the thickness of this link is proportional to the number of genomes in which this is the case, with six distinct classes of link thickness: [1, 10[, [10, 20[, [20, 40[, [60, 80[and [80, +100[genomes.

A

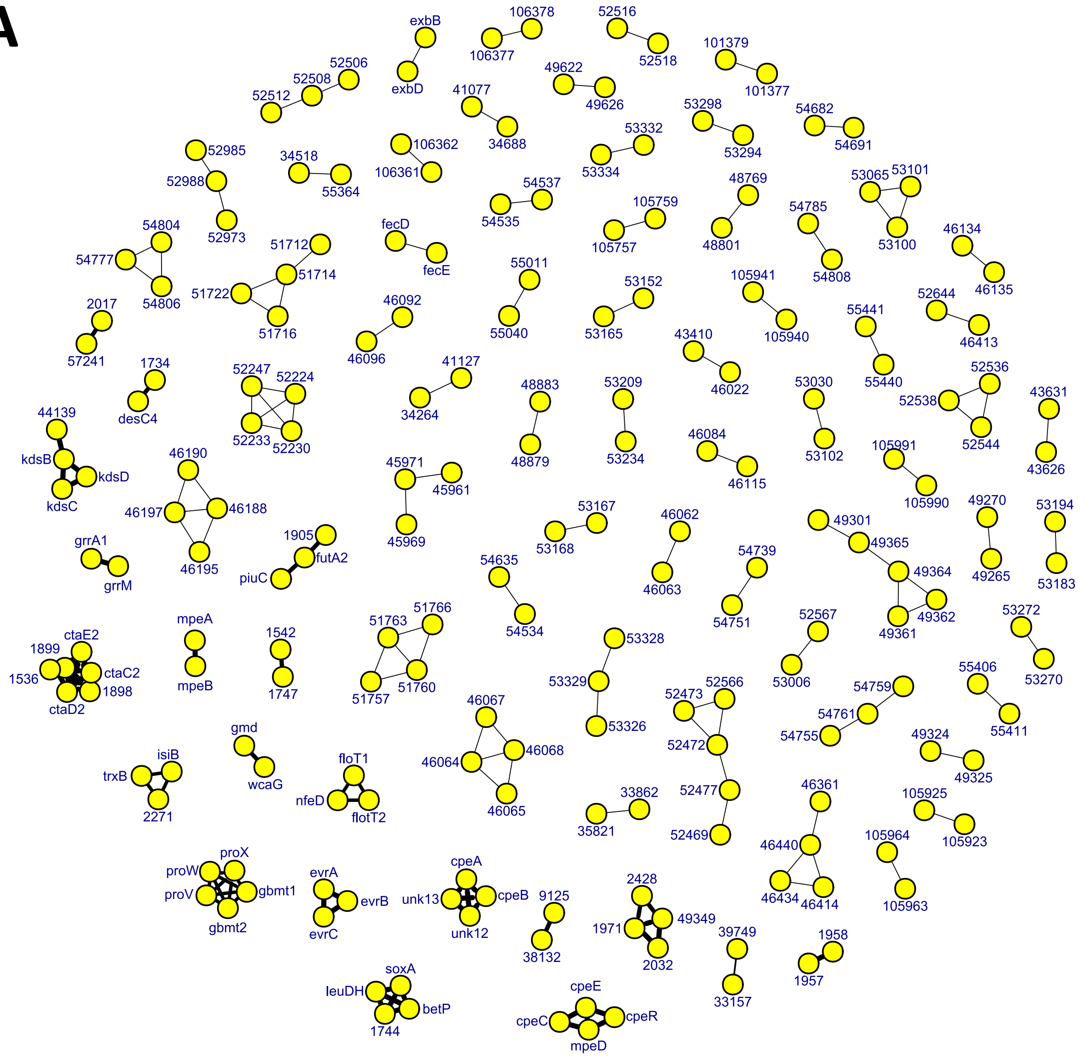

Fig. S4. Same as Fig. S3 for the *Synechococcus* yellow module (*A*)

B

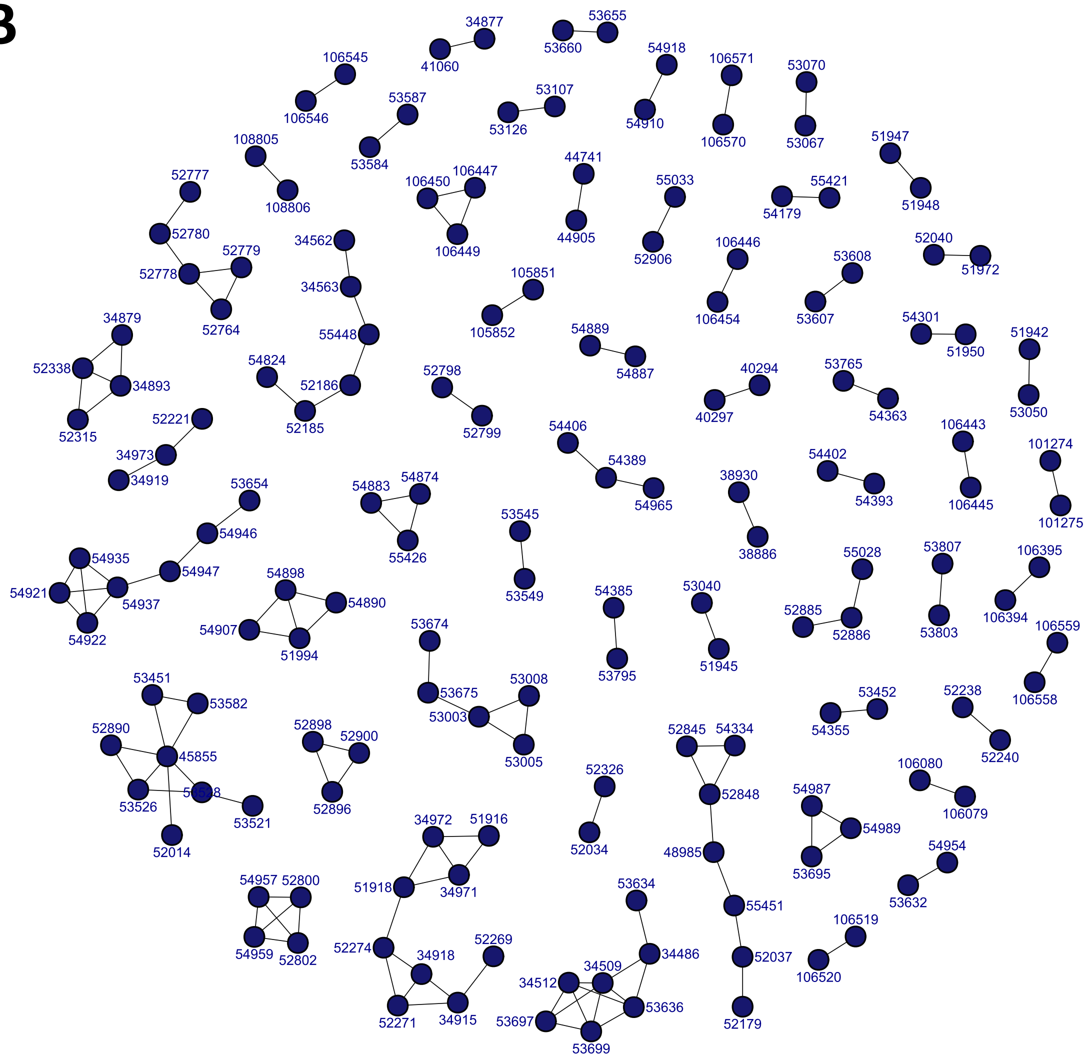

Fig. S4. Continued for the *Synechococcus* midnight blue module (*B*)

C

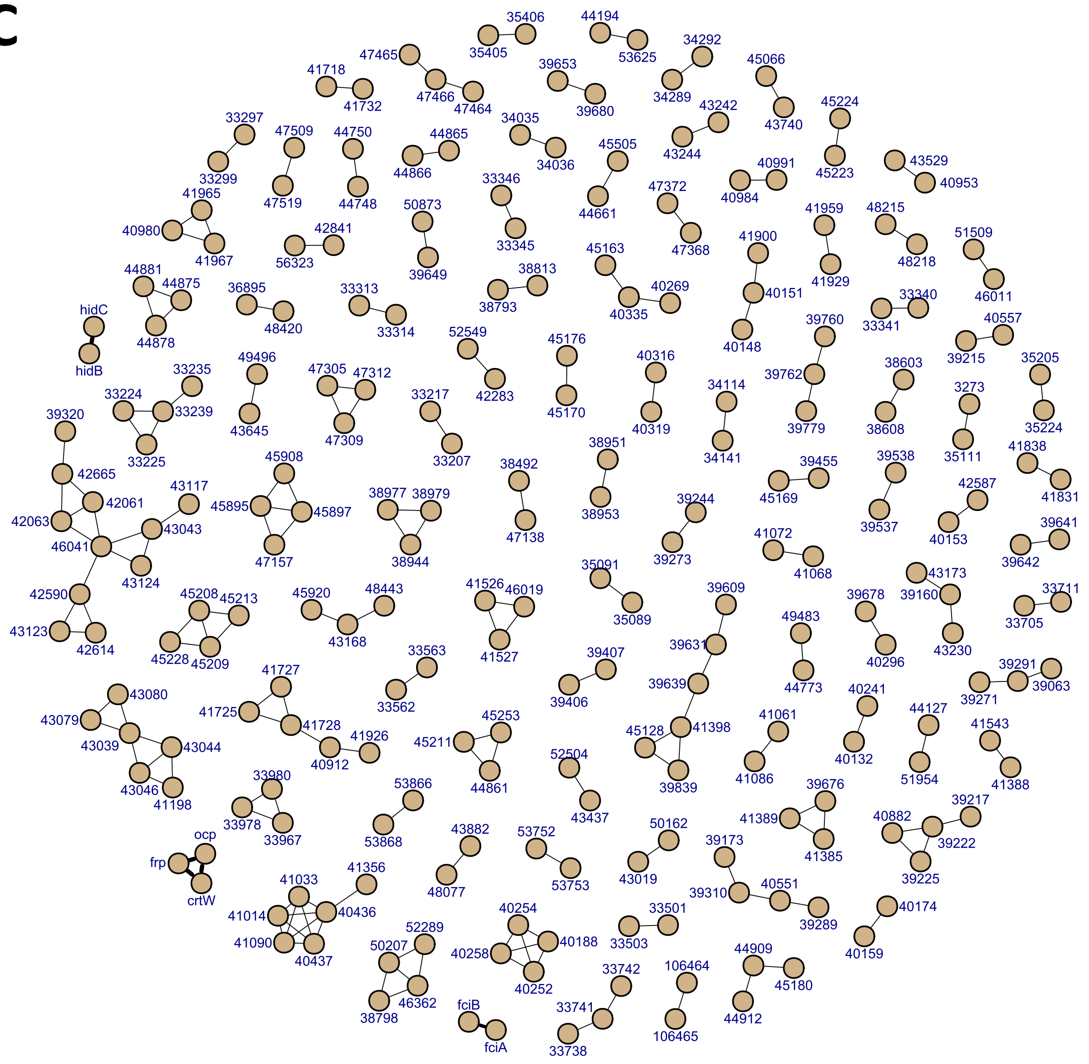

Fig. S4. Continued for the *Synechococcus* tan module (*C*)

D

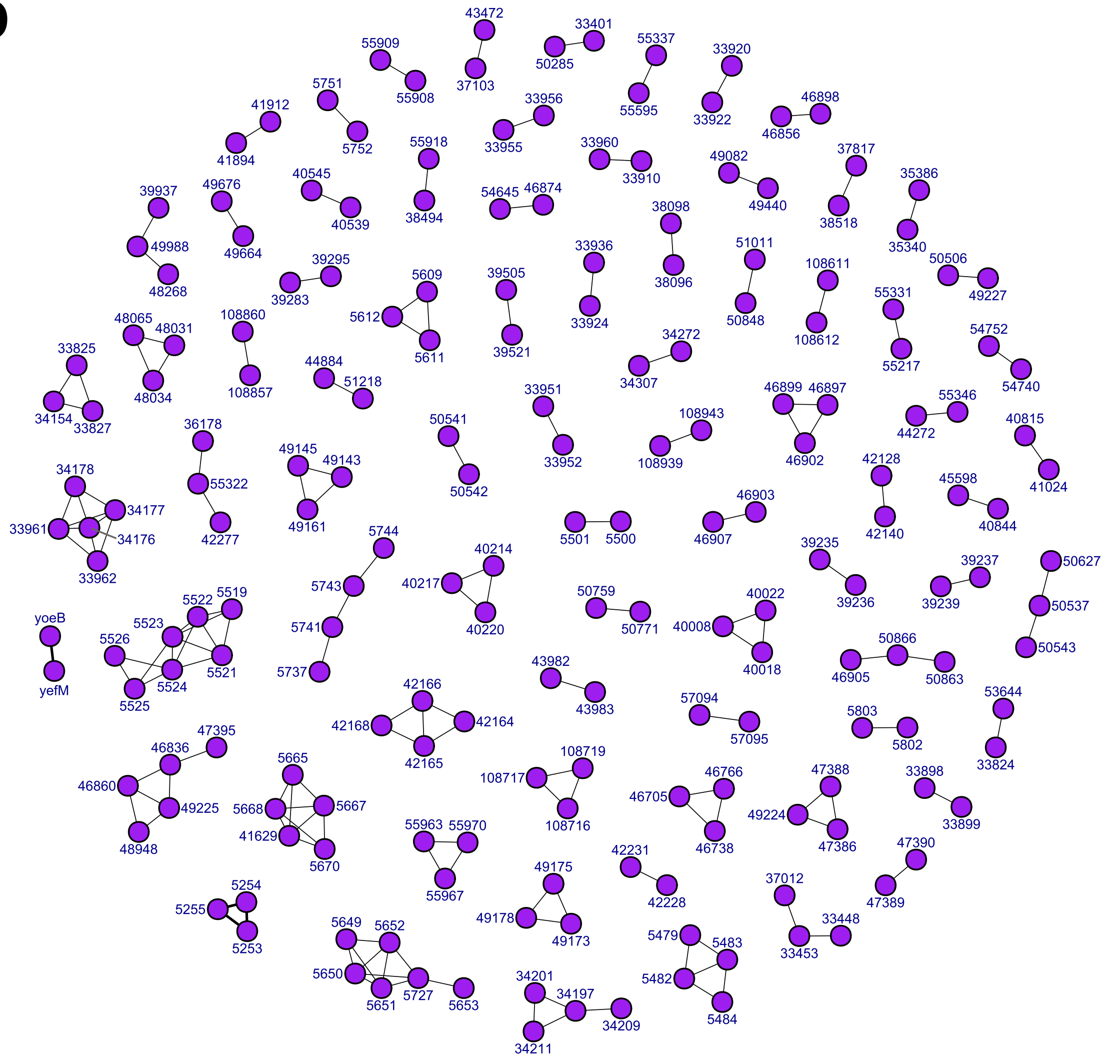

Fig. S4. Continued for the *Synechococcus* purple module (*D*)

E

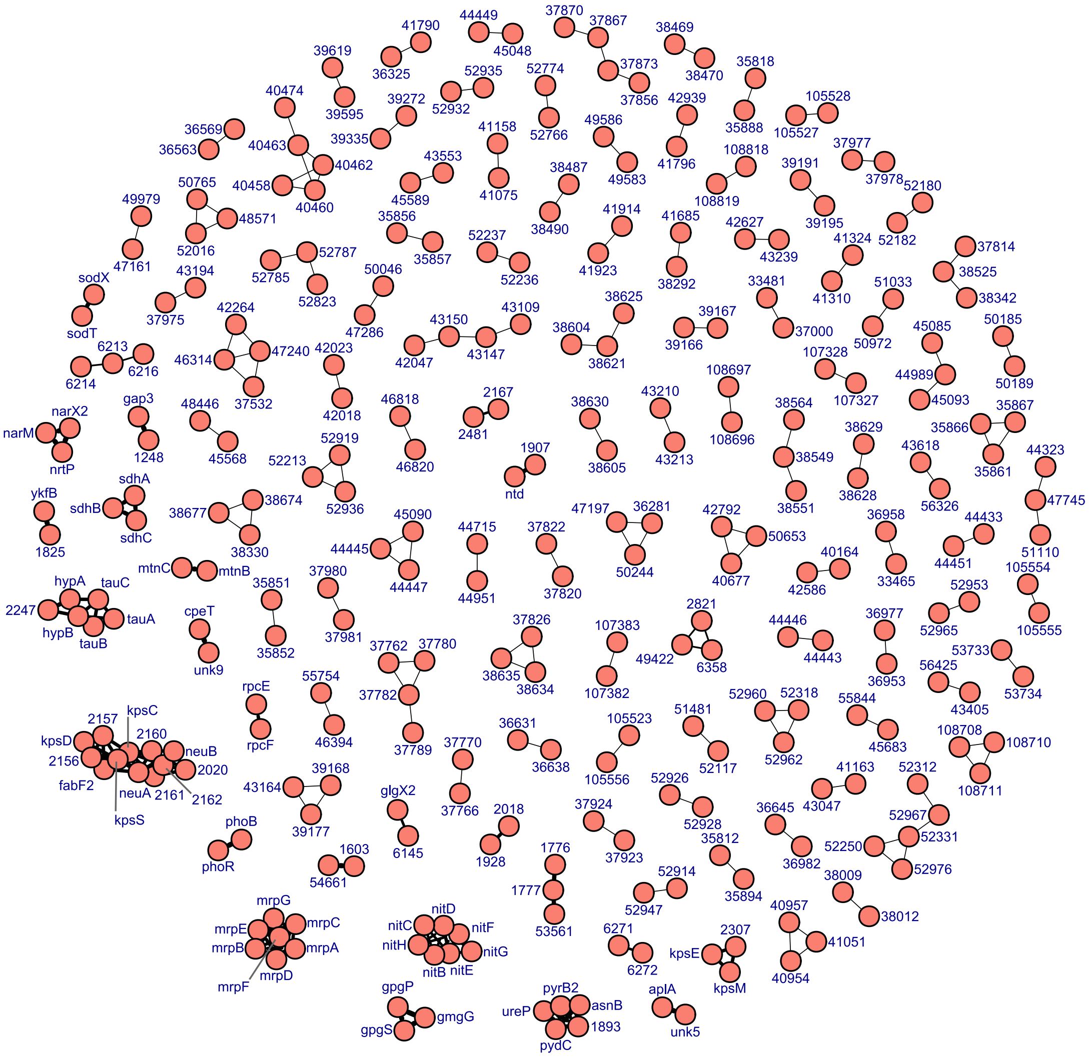

Fig. S4. Continued for the *Synechococcus* salmon module (*E*)

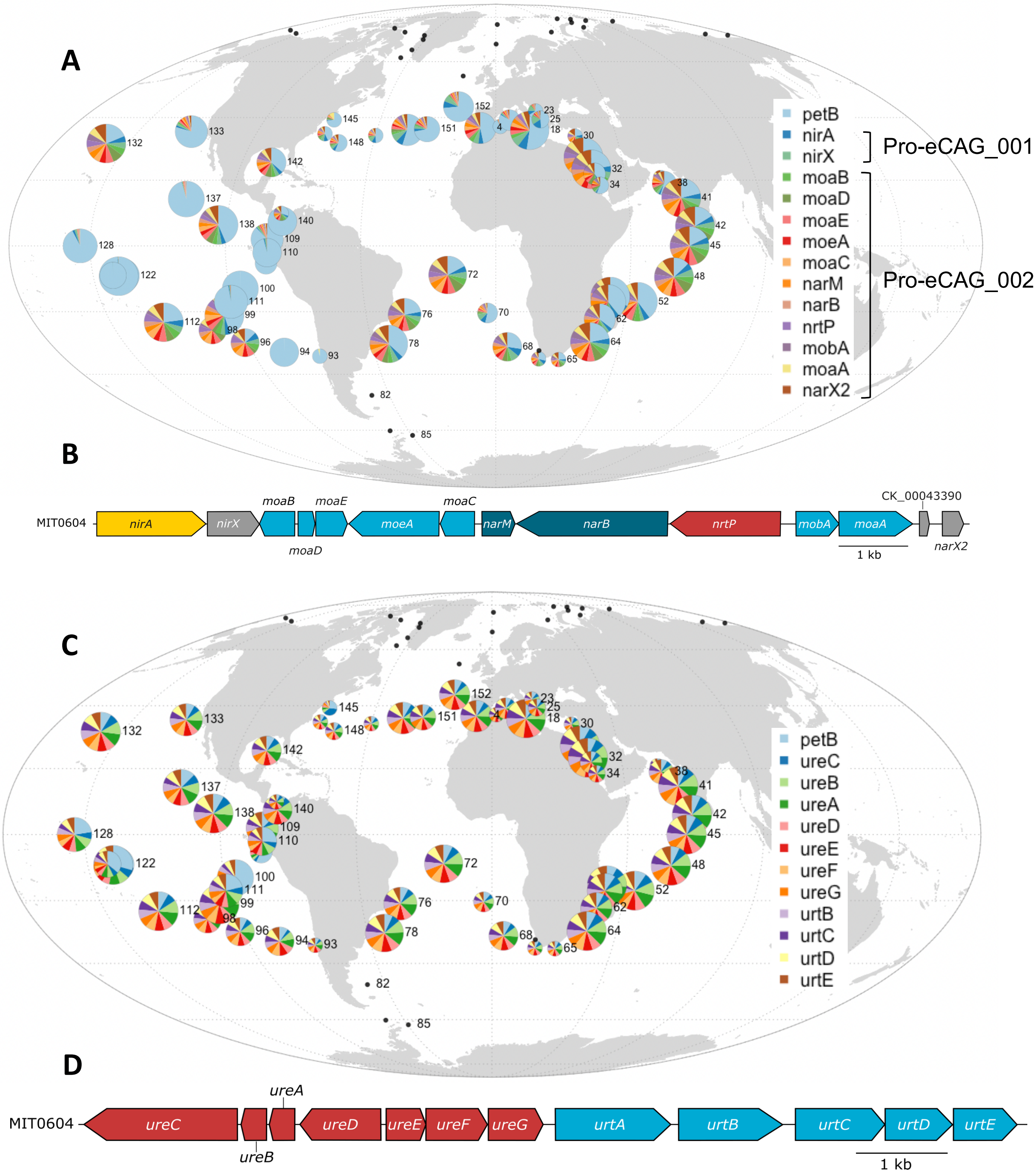

Fig. S5. Global distribution map and genome organization of the *Prochlorococcus* eCAGs involved in the transport and assimilation of inorganic nitrogen and urea. The size of the circle is proportional to the relative abundance of *Prochlorococcus* as estimated based on the single-copy core gene *petB* and this gene was also used to estimate the relative abundance of other genes in the population. (*A*) Pro-eCAG_001 and 002 involved in the transport and assimilation of inorganic nitrogen and (*B*) the corresponding genomic region in *P. marinus* MIT0604. (*C*) Pro-eCAG_003 involved in the transport and assimilation of urea and (D) the corresponding genomic region in *P. marinus* MIT0604. Black dots represent *Tara* Oceans stations for which *Prochlorococcus* read abundance was too low to reach the threshold limit.

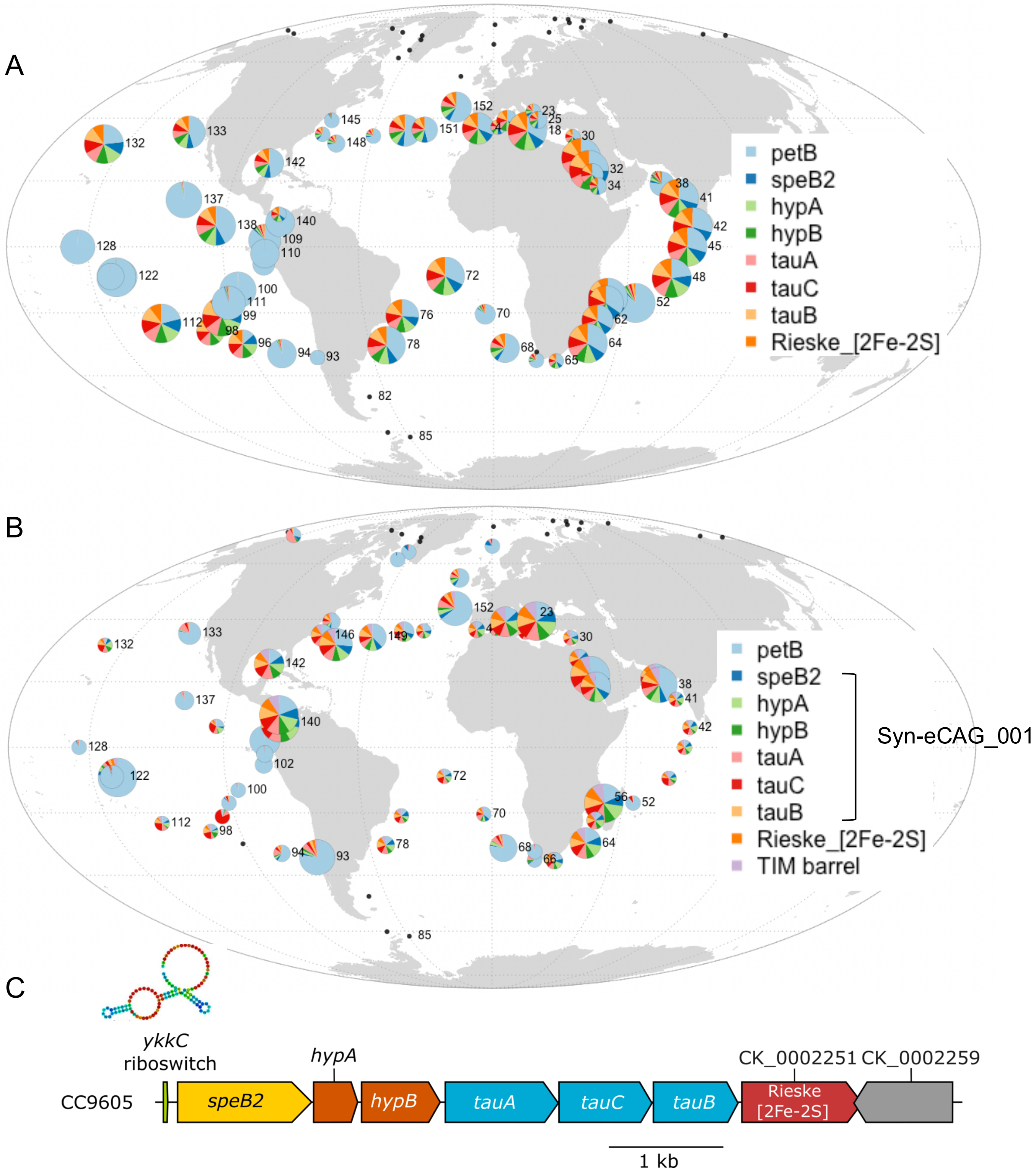

Fig. S6. Global distribution map of the guanidinase eCAG. The size of the circle is proportional to the relative abundance of each genus as estimated based on the single-copy core gene *petB* and this gene was also used to estimate the relative abundance of other genes in the population. (*A*) *Prochlorococcus* Pro-eCAG_004, (*B*) *Synechococcus* Syn-eCAG_001 as well as CK_00002251 and CK_00002259, encoding an iron–sulfur protein and a TIM barrel domain-containing protein, respectively. Note that these two latter genes are not included in Syn-eCAG_001 since they are absent from a few *Synechococcus/Cyanobium* genomes, see Dataset 6). (*C*) Guanidinase gene cluster in *Synechococcus* sp. WH8102 starting with the *ykkC* riboswitch as predicted by regPrecise (https://regprecise.lbl.gov/regulon.jsp?regulon_id=23874). Black dots represent *Tara* Oceans stations for which *Prochlorococcus* or *Synechococcus* read abundance was too low to reach the threshold limit.

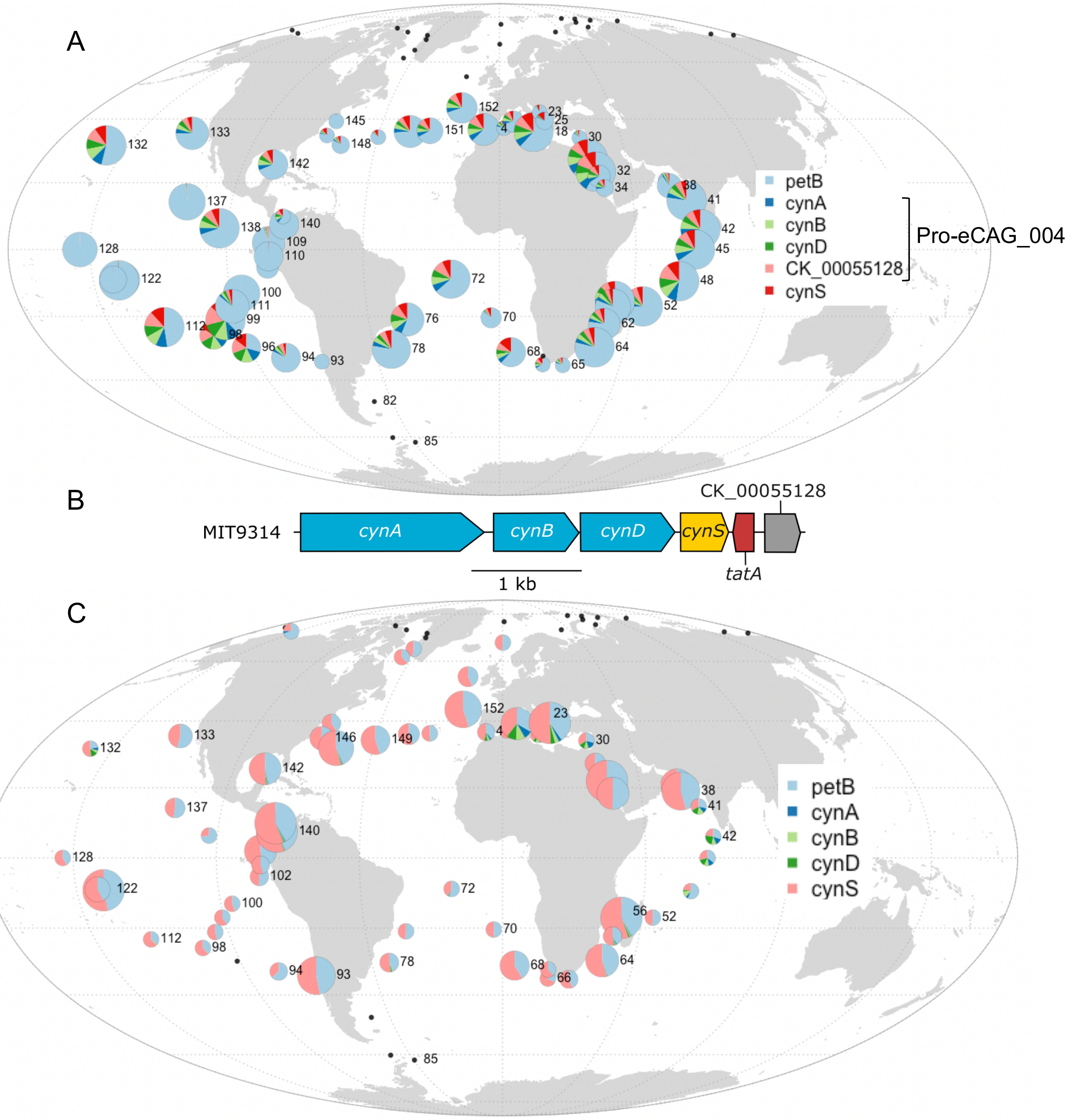

Fig. S7. Global distribution map of picocyanobacterial eCAGs involved in the uptake and degradation of cyanate. The size of the circle is proportional to the relative abundance of each genus as estimated based on the single-copy core gene *petB* and this gene was also used to estimate the relative abundance of other genes in the population. (*A*) *Prochlorococcus* eCAG (Pro-eCAG_005) involved in cyanate transport and uptake. Note that this eCAG does not include *cynS* due to its presence without *cynABD* in several LLI genomes. (*B*) The genomic region in *Prochlorococcus marinus* MIT9314. (*C*) Distribution of the same non-eCAG gene operon in *Synechococcus*. Note that CK_00055128 is absent in *Synechococcus/Cyanobium.* Black dots represent *Tara* Oceans stations for which *Prochlorococcus* or *Synechococcus* read abundance was too low to reach the threshold limit.

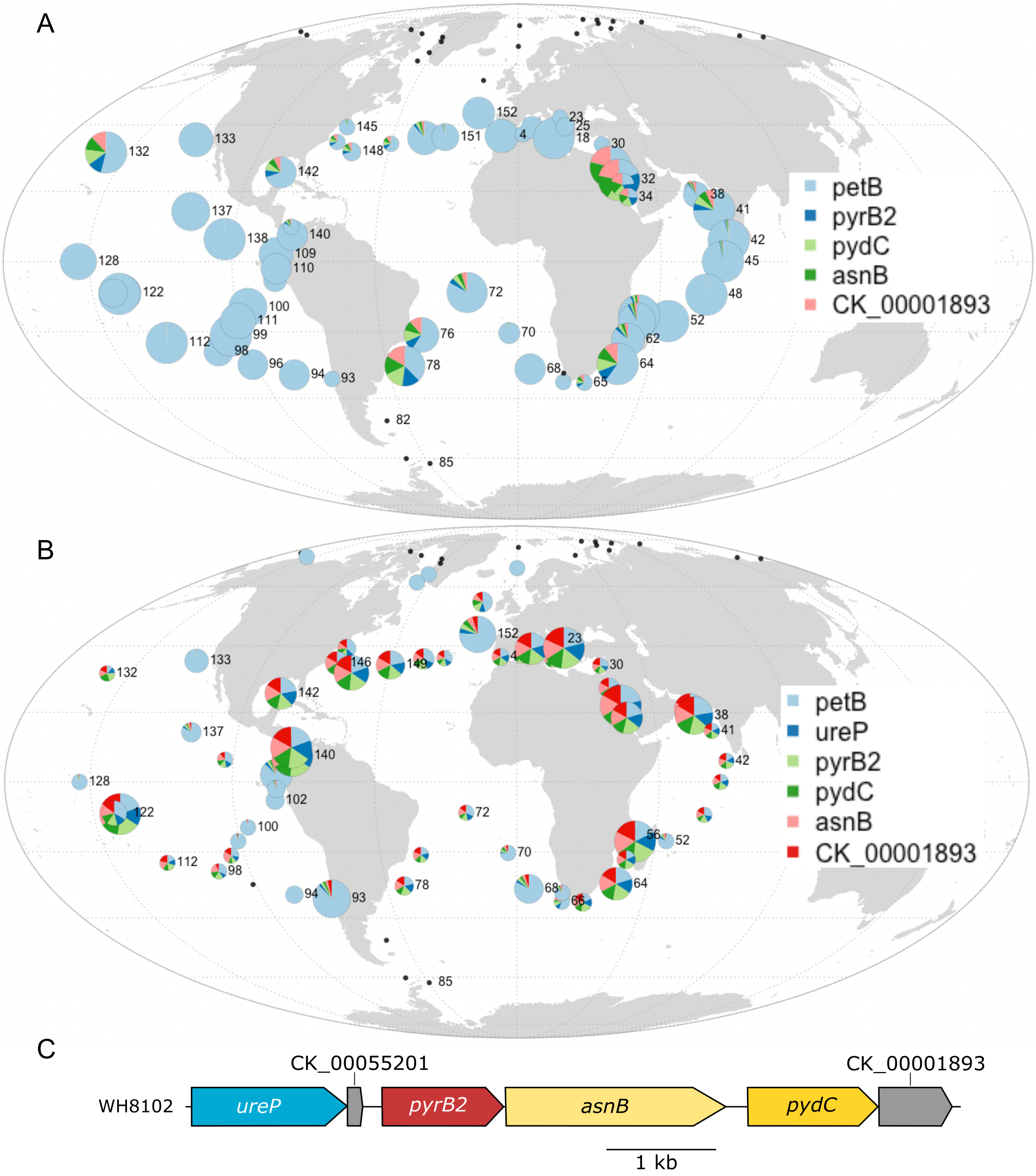

Fig. S8. Global distribution map of picocyanobacterial eCAGs involved in the biosynthesis of pyrimidines. The size of the circle is proportional to the relative abundance of each genus as estimated based on the single-copy core gene *petB* and this gene was also used to estimate the relative abundance of other genes in the population. eCAG involved in the biosynthesis of pyrimidines in (*A*) *Prochlorococcus* (Pro-eCAG_007) and (*B*) *Synechococcus* (Syn-eCAG_003). (*C*) The genomic region in *Synechococcus* sp. WH8102. Black dots represent *Tara* Oceans stations for which *Prochlorococcus* or *Synechococcus* read abundance was too low to reach the threshold limit.

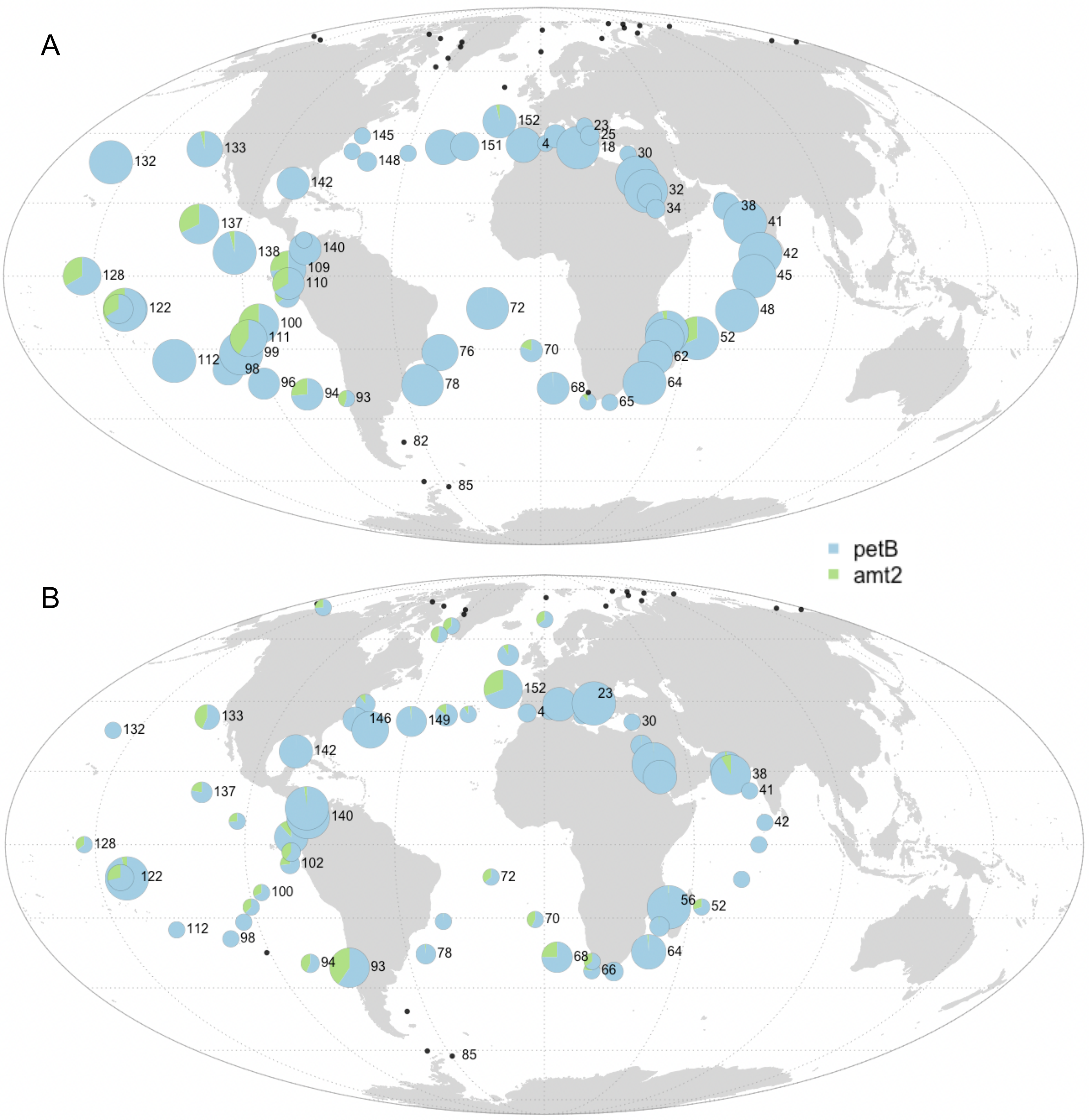

Fig. S9. Global distribution map of the *amt2* gene, potentially involved in ammonium transport. The size of the circle is proportional to the relative abundance of each genus as estimated based on the single-copy core gene *petB* and this gene was also used to estimate the relative abundance of other genes in the population. (*A*) *Prochlorococcus*, (*B*) *Synechococcus.* Black dots represent *Tara* Oceans stations for which *Prochlorococcus* or *Synechococcus* read abundance was too low to reach the threshold limit.

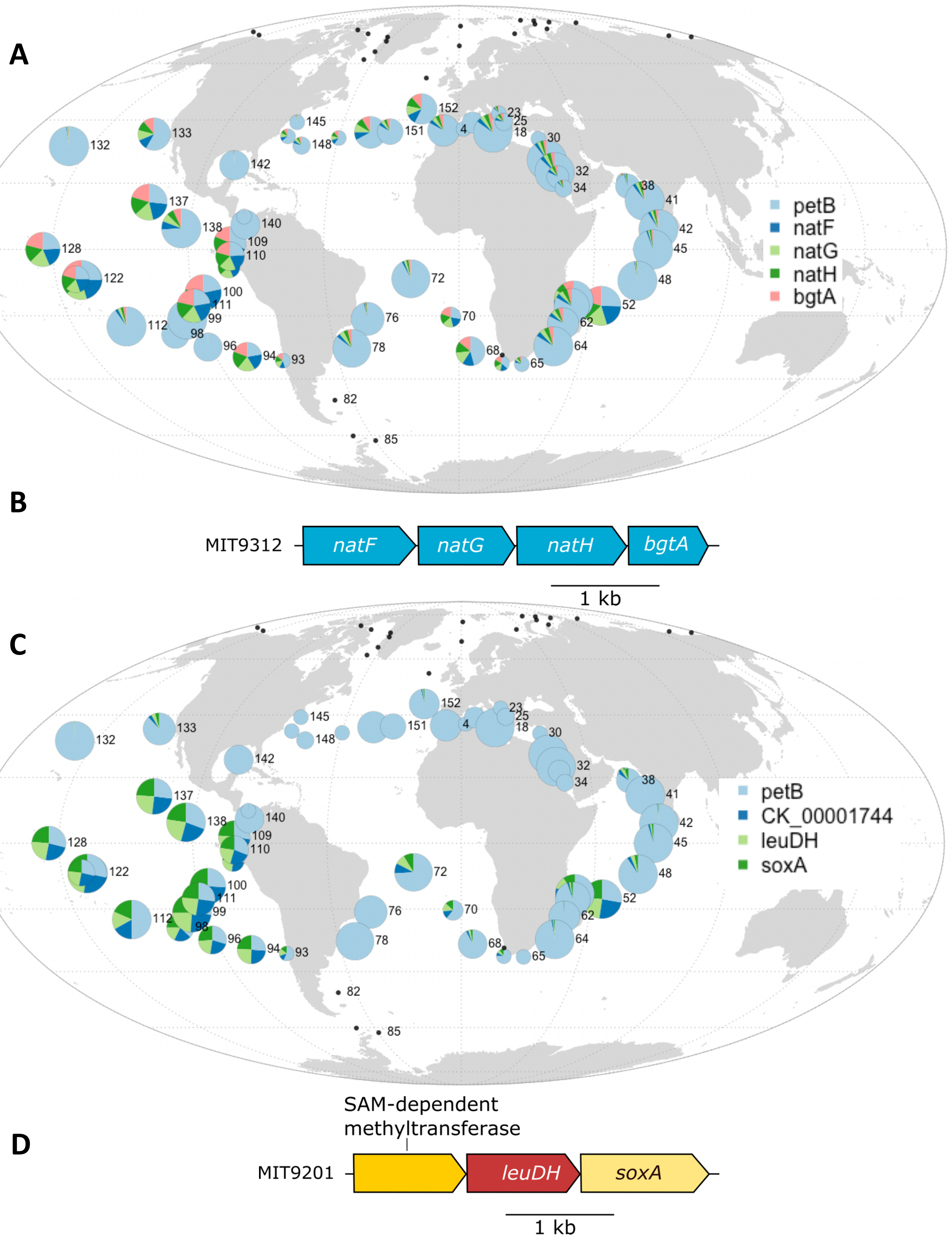

Fig. S10. Global distribution map of the *Prochlorococcus* eCAG putatively involved in amino acid transport or metabolism. The size of the circle is proportional to the relative abundance of *Prochlorococcus* as estimated based on the single-copy core gene *petB* and this gene was also used to estimate the relative abundance of other genes in the population. (*A*) eCAG involved in the ABC-type transport of acidic and neutral polar amino acids (Pro-eCAG_008) and (*B*) the corresponding genomic region in *P. marinus* MIT9312, (*C*) eCAG putatively involved in amino acid metabolism (Pro-eCAG_009) and (*D*) the corresponding genomic region in *P. marinus* MIT9201. Black dots represent *Tara* Oceans stations for which *Prochlorococcus* read abundance was too low to reach the threshold limit.

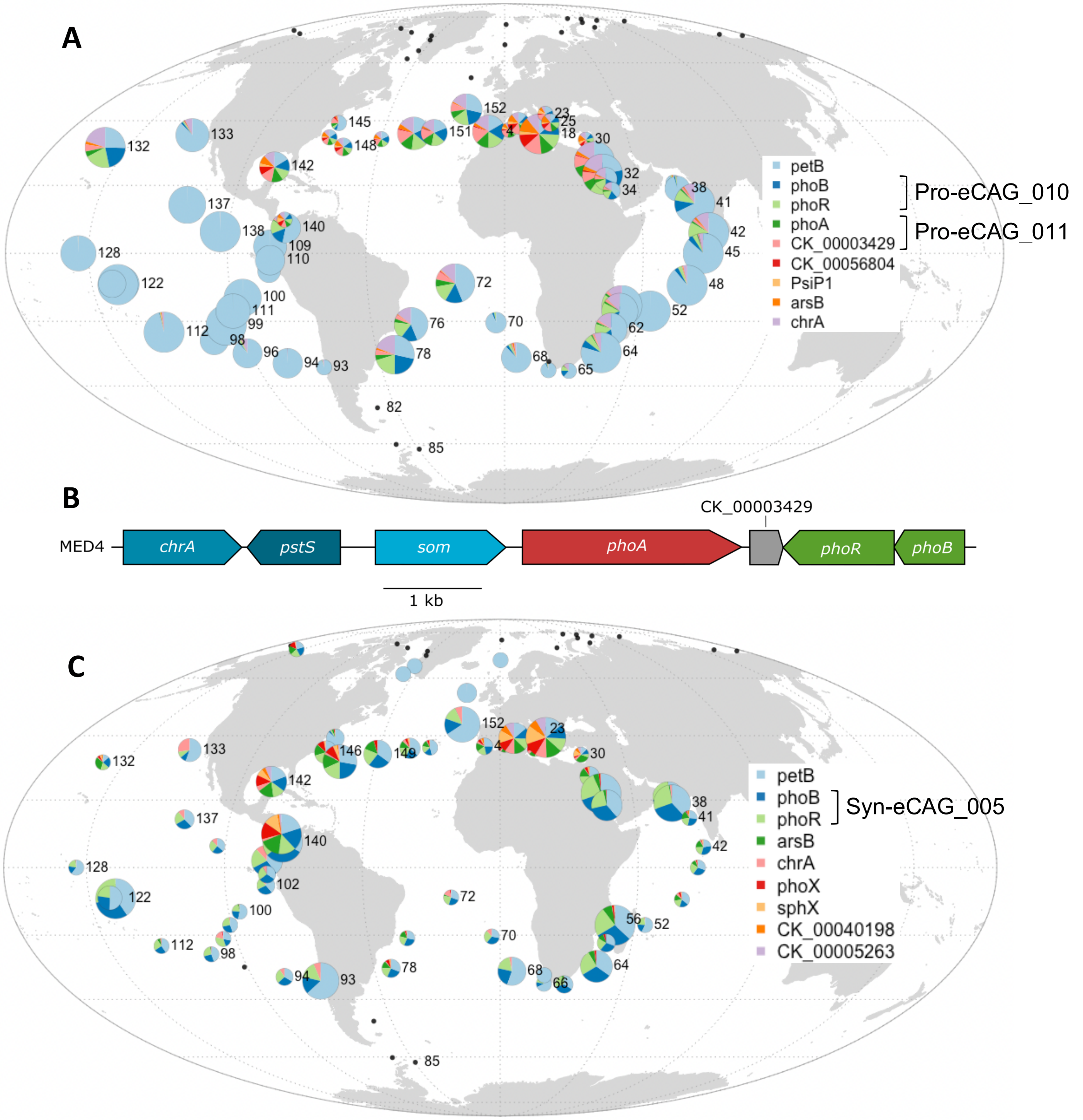

Fig. S11. Global distribution map of picocyanobacterial genes putatively involved in adaptation to P-depletion. The size of the circle is proportional to the relative abundance of each genus as estimated based on the single-copy core gene *petB* and this gene was also used to estimate the relative abundance of other genes in the population. (*A*) *Prochlorococcus* Pro-eCAG_010 and Pro-eCAG_011 as well as genes often retrieved in the same genomic area. (*B*) The corresponding genomic region in *P. marinus* MED4. (*C*) *Synechococcus* Syn-eCAG_005 and marker genes of P-limitation retrieved in the purple module, including CK_00040198 and CK_00052500 encoding putative alkaline phosphatases, both absent from reference *Prochlorococcus* genomes. Black dots represent *Tara* Oceans stations for which *Prochlorococcus* or *Synechococcus* read abundance was too low to reach the threshold limit.

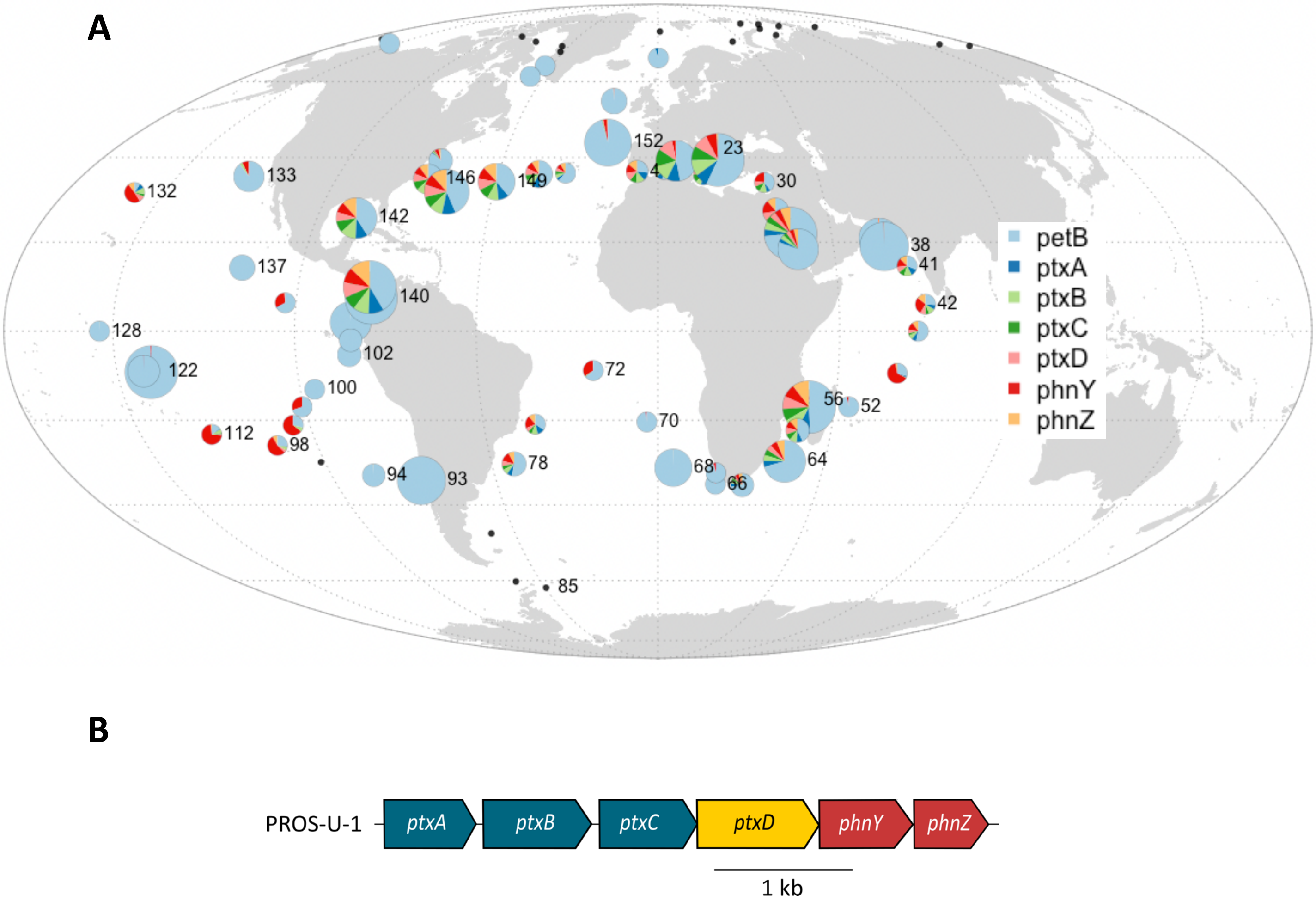

Fig. S12. Global distribution map of *Synechococcus* genes potentially involved in phosphonate and phosphite transport and assimilation. The size of the circle is proportional to the relative abundance of *Synechococcus* as estimated based on the single-copy core gene *petB* and this gene was also used to estimate the relative abundance of other genes in the population. (*A*) Global distribution map and (*B*) the corresponding genomic region in *Synechococcus* sp. PROS-U-1. Black dots represent *Tara* Oceans stations for which *Synechococcus* read abundance was too low to reach the threshold limit.

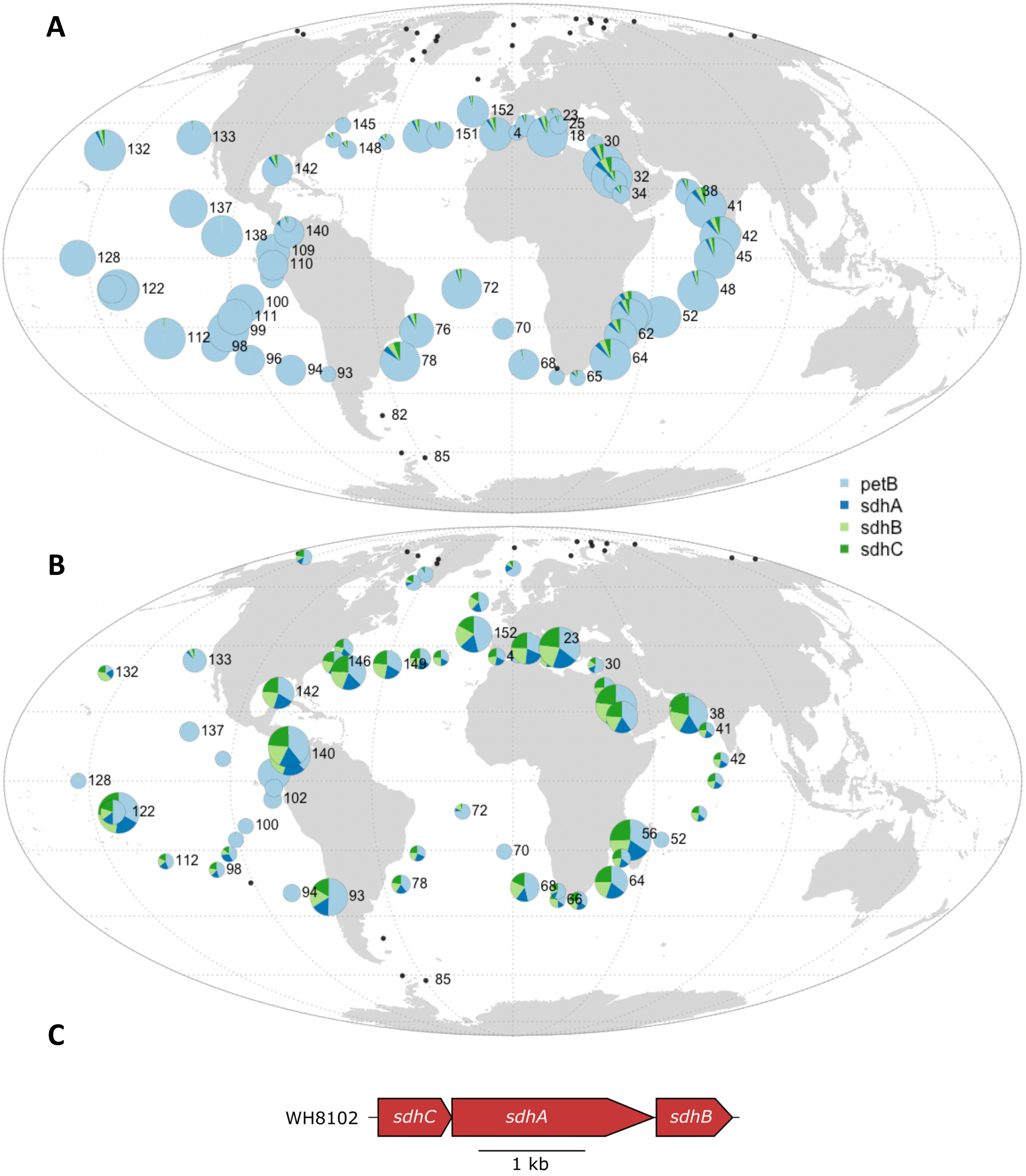

Fig. S13. Global distribution map of eCAGs involved in the biosynthesis of succinate dehydrogenase. The size of the circle is proportional to the relative abundance of each genus as estimated based on the single-copy core gene *petB* and this gene was also used to estimate the relative abundance of other genes in the population. (*A*) *Prochlorococcus* Pro-eCAG_014, (*B*) *Synechococcus* Syn-eCAG_006 and (*C*) the corresponding genomic region in *Synechococcus* sp. WH8102. Black dots represent *Tara* Oceans stations for which *Prochlorococcus* or *Synechococcus* read abundance was too low to reach the threshold limit.

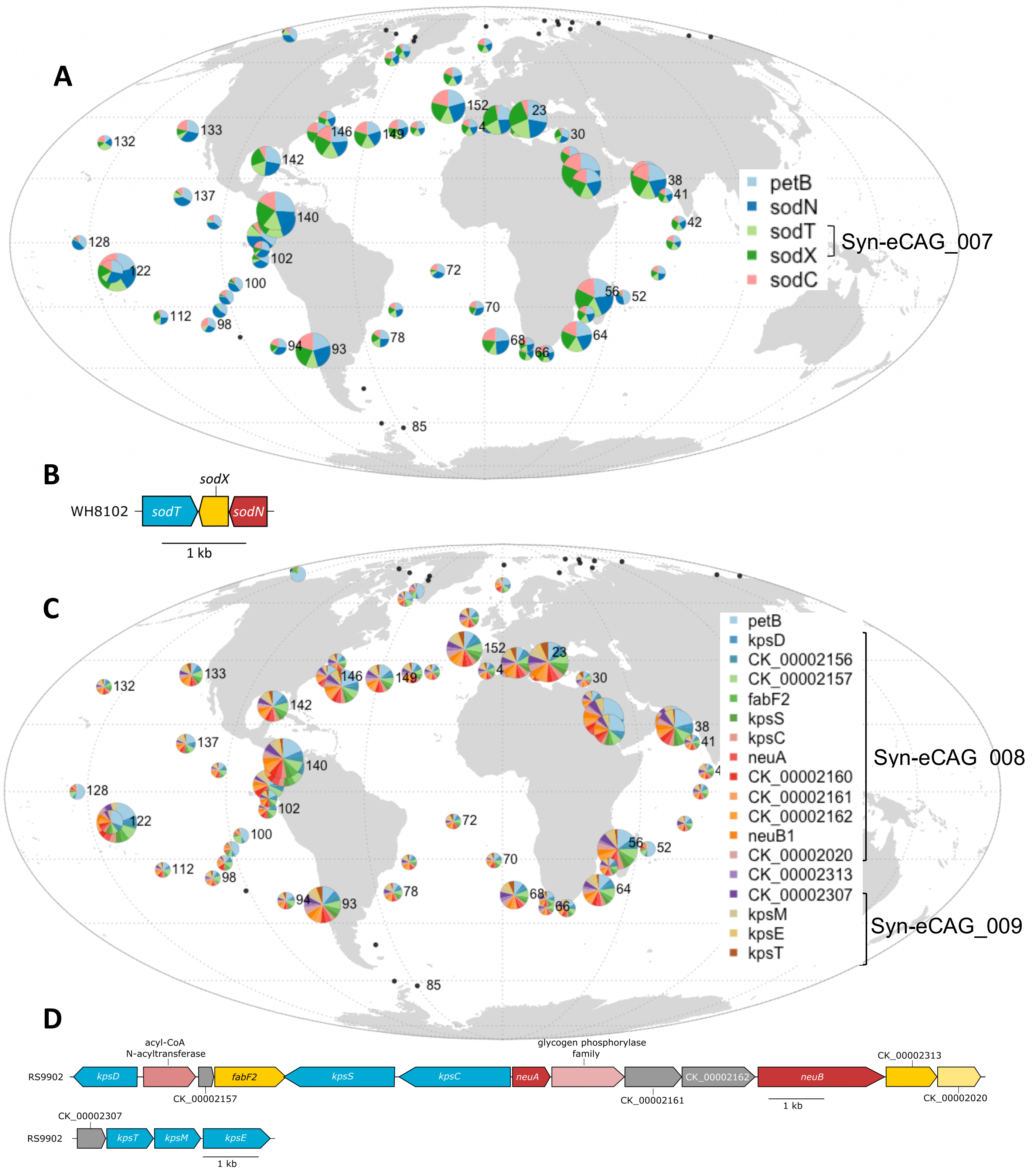

Fig. S14. Global distribution map of *Synechococcus* eCAGs specifically enriched in Fe-replete areas. The size of the circle is proportional to the relative abundance of *Synechococcus* as estimated based on the single-copy core gene *petB* and this gene was also used to estimate the relative abundance of other genes in the population. (*A*) Syn-eCAG_008 encompassing two genes related to nickel transport (*sodT*) and maturation (*sodX*) of the Ni-superoxide dismutase (*sodN*), the latter being also shown for comparison. (*B*) The corresponding genomic region in *Synechococcus sp.* WH8102 (*C*) Syn-eCAG_009 and other related gene putatively involved in the biosynthesis of polysaccharide capsules. (D) The corresponding genomic region in *Synechococcus* sp. RS9902. Black dots represent *Tara* Oceans stations for which *Synechococcus* read abundance was too low to reach the threshold limit.

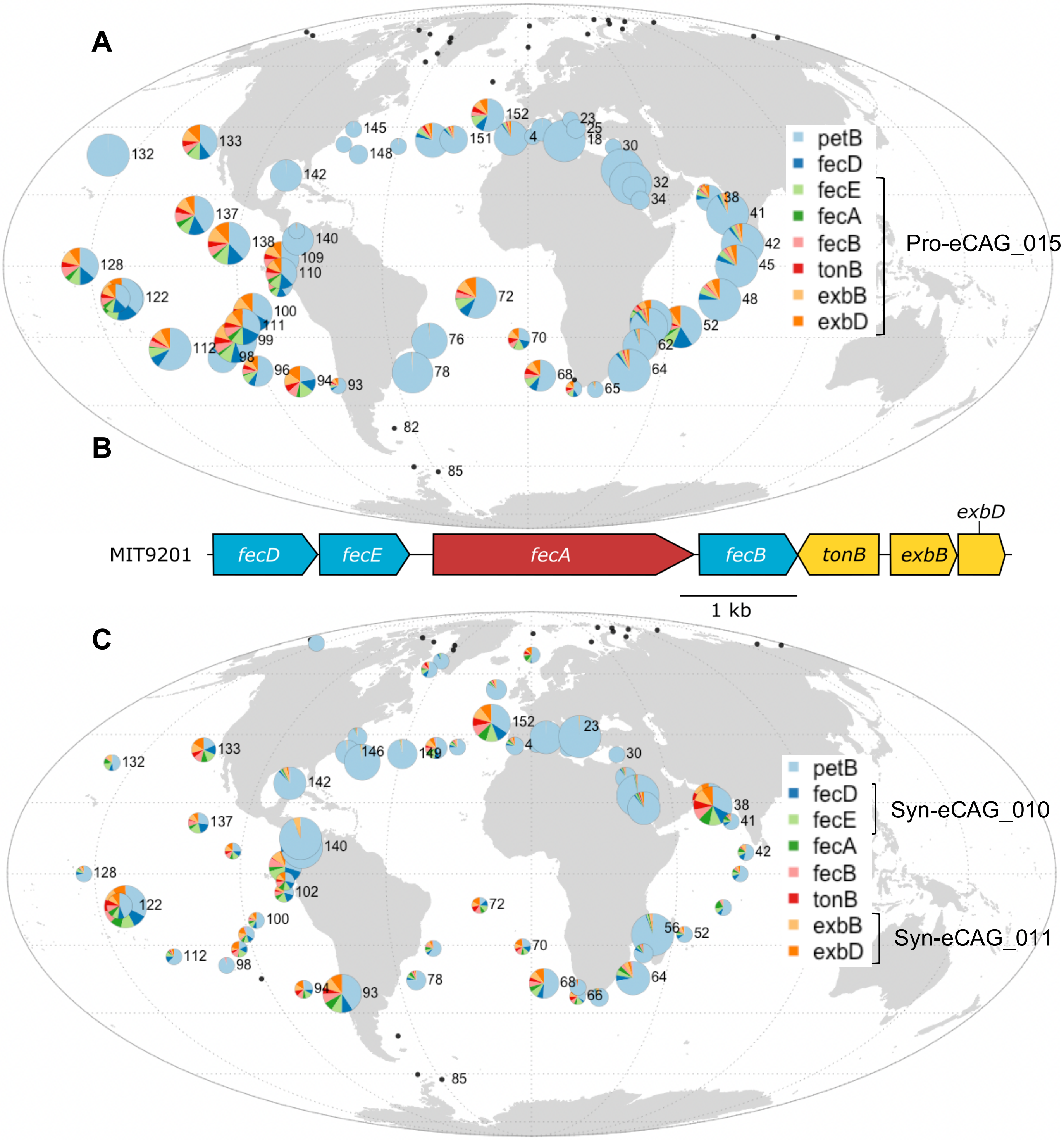

Fig. S15. Global distribution map of eCAGs involved in TonB-dependent siderophore uptake. The size of the circle is proportional to the relative abundance of each genus as estimated based on the single-copy core gene *petB* and this gene was also used to estimate the relative abundance of other genes in the population. (*A*) *Prochlorococcus* Pro-eCAG_015 and (*B*) the corresponding genomic region in *P. marinus* MIT9201. (*C*) *Synechococcus* Syn-eCAG_010 and 011**.** Black dots represent *Tara* Oceans stations for which *Prochlorococcus* or *Synechococcus* read abundance was too low to reach the threshold limit.

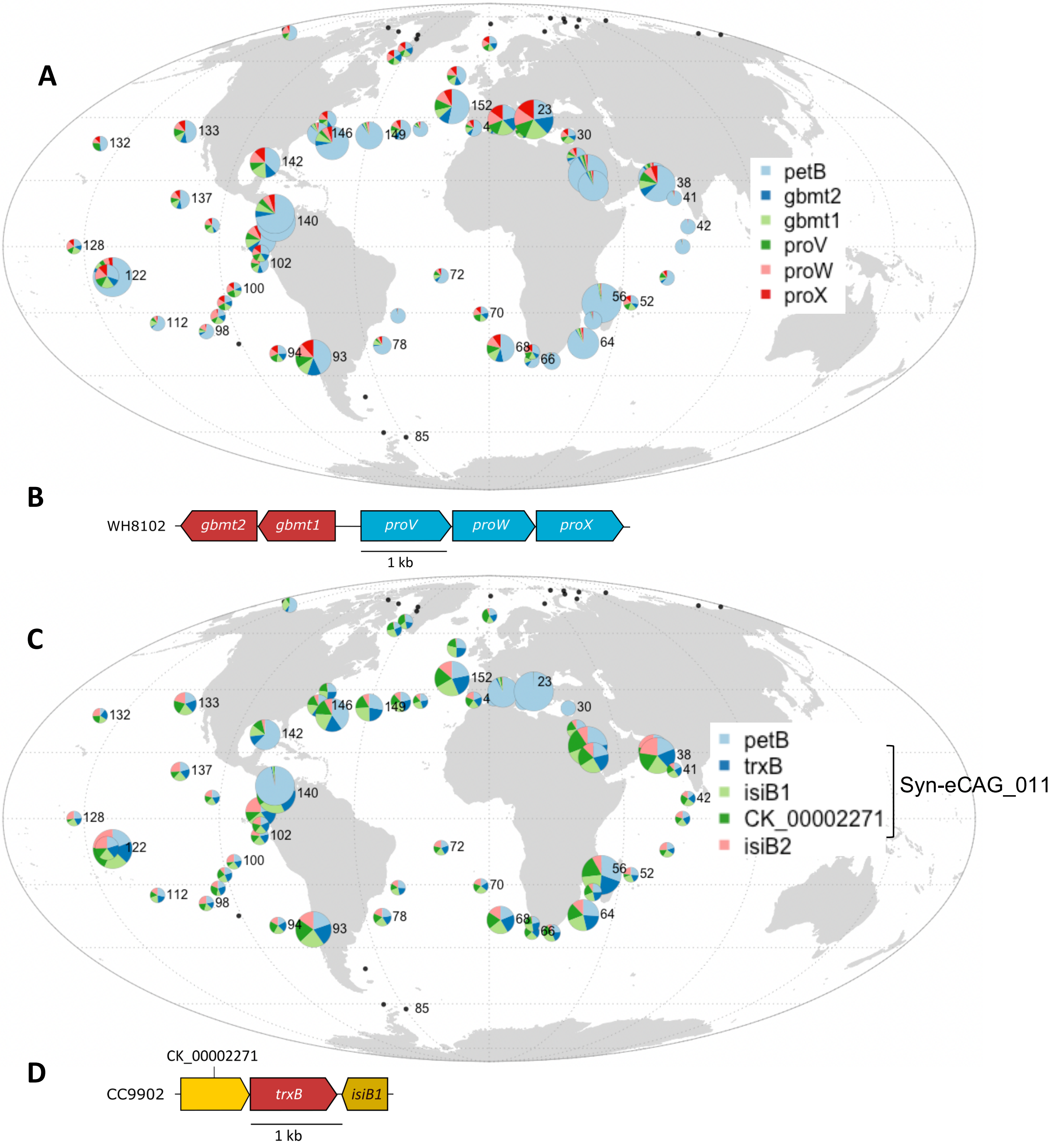

Fig. S16. Global distribution map of the *Synechococcus* eCAGs enriched in Fe-depleted areas. The size of the circle is proportional to the relative abundance of *Synechococcus* as estimated based on the single-copy core gene *petB* and this gene was also used to estimate the relative abundance of other genes in the population. (*A*) Syn-eCAG_010 involved in glycine betaine synthesis and transport and (*B*) the corresponding genomic region in *Synechococcus* sp. WH8102. (*C*) Syn-eCAG_011 encoding a flavodoxin and a thioredoxin reductase and (*D*) the corresponding genomic region in *Synechococcus* sp. CC9902. The second *isiB* copy (*isiB2*) is shown here for comparison. Black dots represent *Tara* Oceans stations for which *Synechococcus* read abundance was too low to reach the threshold limit.

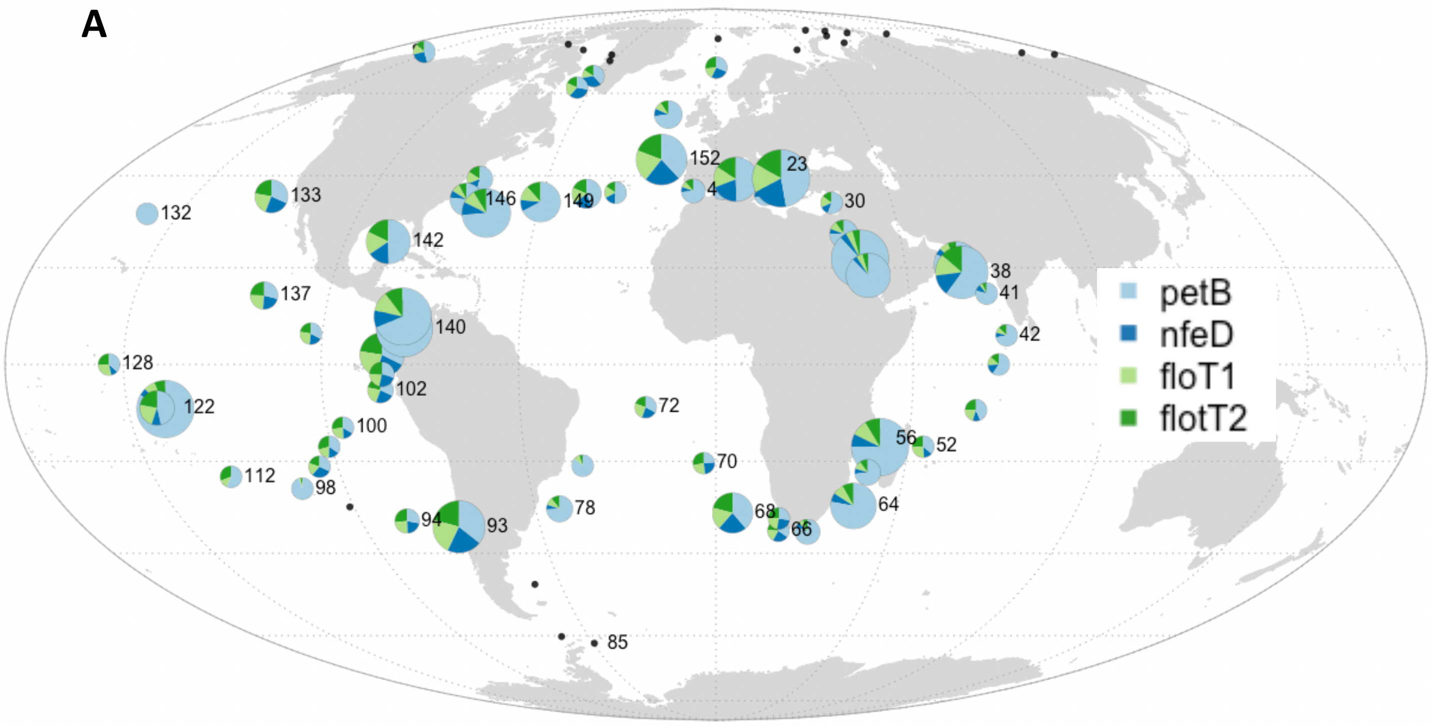

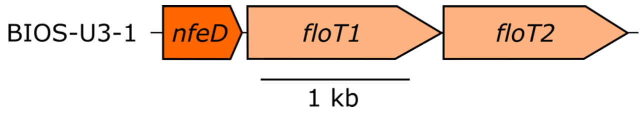

Fig. S17. Global distribution map of the *Synechococcus* eCAGs enriched in Fe-depleted areas (continued). (*A*) Syn-eCAG_012 involved in the production of lipid rafts and (*B*) the corresponding genomic region in *Synechococcus* sp. BIOS-U3-1**.** The size of the circle is proportional to the relative abundance of *Synechococcus* as estimated based on the single-copy core gene *petB* and this gene was also used to estimate the relative abundance of other genes in the population. Black dots represent *Tara* Oceans stations for which *Synechococcus* read abundance was too low to reach the threshold limit.

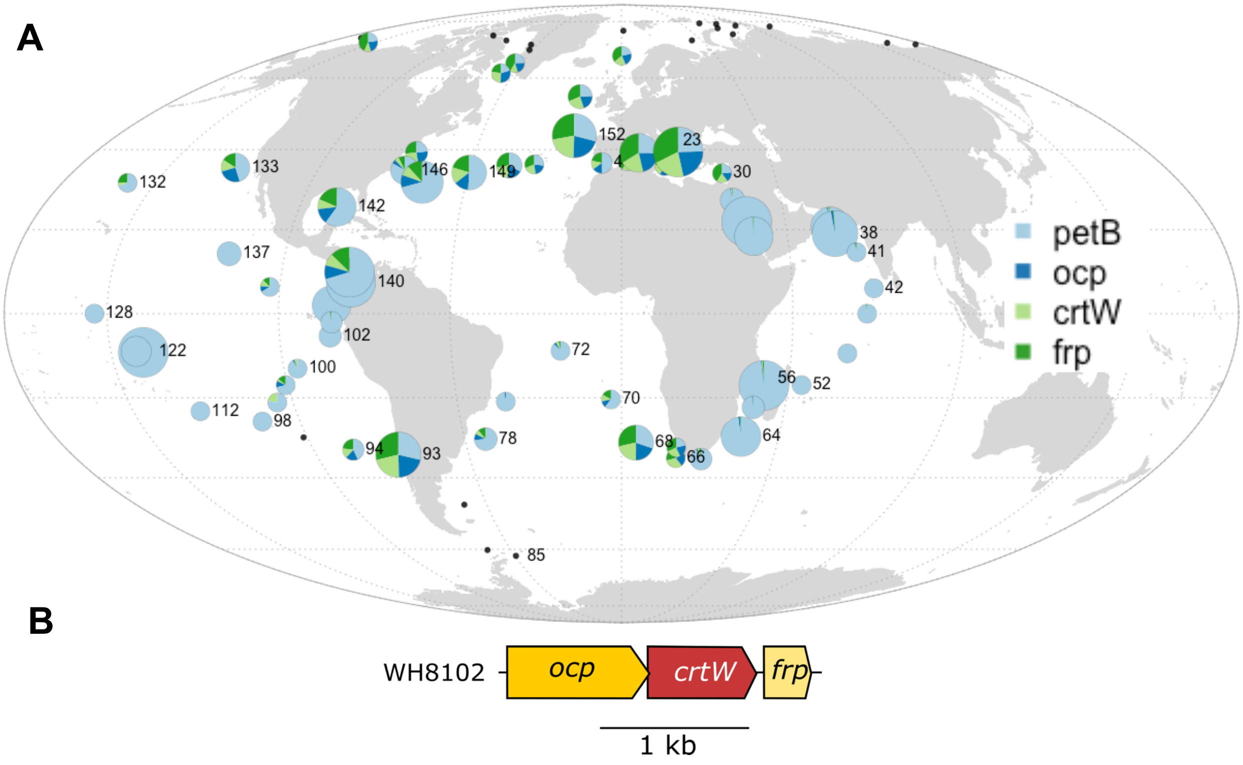

Fig. S18. Global distribution map of a *Synechococcus* eCAG enriched in cold waters. (*A*) Syn-eCAG_016 involved in orange caroteno-protein mediated photoprotection and (*B*) the corresponding genomic region in *Synechococcus* sp. WH8102. The size of the circle is proportional to the relative abundance of *Synechococcus* as estimated based on the single-copy core gene *petB* and this gene was also used to estimate the relative abundance of other genes in the *Synechococcus* population. Black dots represent *Tara* Oceans stations for which *Synechococcus* read abundance was too low to reach the threshold limit.

SI References

1. Sunagawa S, Coelho LP, Chaffron S, Kultima JR, Labadie K, Salazar G, et al. Structure and function of the global ocean microbiome. Science 2015; 348: 1261359–1261359.
2. Farrant GK, Doré H, Cornejo-Castillo FM, Partensky F, Ratin M, Ostrowski M, et al. Delineating ecologically significant taxonomic units from global patterns of marine picocyanobacteria. Proc Natl Acad Sci USA 2016; 113: E3365–E3374.
3. Doré H, Farrant GK, Guyet U, Haguait J, Humily F, Ratin M, et al. Evolutionary mechanisms of long-term genome diversification associated with niche partitioning in marine picocyanobacteria. Front Microbiol 2020; 11.
4. Zhang B, Horvath S. A general framework for weighted gene co-expression network analysis. Stat Appl Genet Mol Biol 2005; 4: Article17.
5. Szmrecsanyi B Grammatical Variation in British English Dialects: A Study in Corpus-Based Dialectometry. 2012. Cambridge University Press, Cambridge.
6. Oksanen J, Blanchet FG, Kindt R, Legendre P, Minchin PR, O'Hara RB, et al. Vegan: Community Ecology Package. R package Version 2.4-3. 2017.
7. Langfelder P, Horvath S. WGCNA: an R package for weighted correlation network analysis. BMC Bioinfo 2008; 9: 559.
8. Frick A, Ludwig A, Mehldau H: A Fast Adaptive Layout Algorithm for Undirected Graphs, Proc. Graph Drawing 1994, LNCS 894, pp. 388-403, 1995.
9. Fruchterman TMJ and Reingold EM (1991). Graph Drawing by Force-directed Placement. Software - Practice and Experience, 21(11):1129-1164.
10. Csardi G, Nepusz T (2006). The igraph software package for complex network research, InterJournal, Complex Systems 1695. https://igraph.org.
